# Potential for Regional Resilience to Ocean Warming and Acidification Extremes: Projected Vulnerability Under Contrasting Pathways and Thresholds

**DOI:** 10.1111/gcb.70360

**Published:** 2025-07-21

**Authors:** Elise M. Olson, Jasmin G. John, John P. Dunne, Charles A. Stock, Elizabeth J. Drenkard

**Affiliations:** ^1^ Atmospheric and Oceanic Sciences Princeton University Princeton New Jersey USA; ^2^ Canadian Centre for Climate Modelling and Analysis Environment and Climate Change Canada Victoria British Columbia Canada; ^3^ NOAA/OAR/Atlantic Oceanographic and Meteorological Laboratory Miami Florida USA; ^4^ NOAA/OAR/Geophysical Fluid Dynamics Laboratory Princeton New Jersey USA

**Keywords:** adaptation, climate change, extreme events, multiple ecosystem stressors, ocean acidification, ocean warming

## Abstract

We analyze the frequency and amplitude of projected warming and ocean acidification extremes under high CO_2_ and strongly mitigating scenarios. We find interpretational differences in projections arising from methodological choices associated with specification of stressor thresholds. Use of absolute versus distribution‐based thresholds, and, in the distribution‐based case, the inclusion or exclusion of seasonal variability, can lead to very different regional patterns in projected stress. The choice of fixed versus adaptive baseline, for example, determines whether future stress frequency in the low‐CO_2_ scenario most closely resembles that in the high‐emissions scenario or historical period. We find that mitigation through emissions reductions, in combination with representation of rates of adaptation that are realistic for some marine organisms, has the potential to dampen end of century threshold exceedance to frequencies of occurrence closer to the recent historical period than to the high‐emissions scenario.

## Graphical abstract

We analyze the frequency and amplitude of projected warming and ocean acidification extremes under a high and a lower, strongly mitigating, CO_2_ scenario and find interpretational differences arising from choices associated with the specification of stressor thresholds. The choice of fixed versus adaptive baseline determines whether future stress frequency in the low‐CO_2_ scenario most closely resembles the high‐emissions scenario or the historical period. Mitigation through emissions reductions, combined with the representation of rates of adaptation realistic for some marine organisms, has the potential to dampen end‐of‐century threshold exceedance to frequencies closer to the recent historical period than to the high‐emissions scenario.

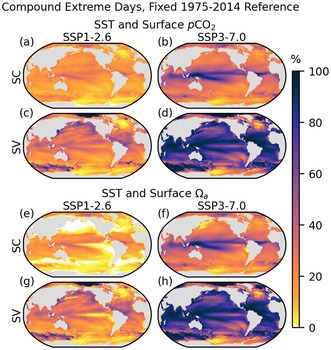

## 1 Introduction

As climate change shifts temperature and ocean acidification conditions beyond the recent historical levels under which modern ecosystems have emerged, ecosystem impacts are anticipated. Ecosystem changes could be deleterious to human food security, economic, and cultural interests. Regions of overlap of extreme change in the mean states and seasonal cycles of multiple ecosystem stressors have been identified and assessed under a range of climate projections (Bopp et al. 2013; Kwiatkowski et al. 2020).

The projected extent and frequency of various types of extreme events with potential ecosystem stress impacts, individually and in combination, have also become a focus for research. Gruber et al. (2021) outlined many of the methodological choices in extreme event definition, including relative, distribution‐based versus absolute thresholds; fixed versus shifting reference baseline; consideration of seasonally varying or deseasonalized data; and whether to include additional criteria such as minimum duration. In this context “absolute” refers to thresholds fixed at a specific level of some environmental variable, usually motivated by physical, chemical, or physiological constraints; an example is an aragonite saturation state ($\Omega_{a}$) threshold of 1, distinguishing between corrosive, under‐saturated environmental conditions and supersaturation with respect to aragonite, a mineral form of calcium carbonate produced by and serving a structural function for some marine organisms, including coral. A shifting baseline may be applied to the definition of relative thresholds with respect to a non‐stationary distribution, typically reflecting a hypothesis that organisms may have the capacity to adapt to gradual change in the mean environmental state but experience stress in response to unaccustomed deviations from that mean state. The importance of clearly signaling these choices in the context of warming and marine heatwaves was highlighted by Amaya et al. (2023), pointing to the need for clarity to support coastal adaptation and resource management, with the understanding that no single approach can be anticipated to represent the responses of all marine organisms, ecosystems, or locations.

Many recent studies have focused on the frequency of temperature and/or acidity extreme events surpassing percentile‐based relative thresholds (Burger et al. 2022; Gruber et al. 2021; Hauri et al. 2024; Hobday et al. 2016; Le Grix et al. 2022). Association of these events with ecosystem stress posits that organisms are well‐adapted to conditions that have been historically commonplace in their local habitat, and that stress is induced by increasing exposure to previously rare conditions. Within this framework, organisms adapted to highly variable environments are anticipated to exhibit greater tolerance to environmental stress, a hypothesis with growing support, particularly in the context of thermal tolerance (Cooley et al. 2022; Sunday et al. 2019), though not applicable across the board to all organisms and ecosystems.

Additionally, within this distribution‐based framework, many authors have chosen to define event extremity relative to the seasonal variability, i.e., with thresholds specific to the time of year, such that extreme events occur throughout the year. An unusually warm winter or spring, for example, may have adverse consequences despite temperatures being cooler than those experienced in summer. For instance, unseasonably warm conditions could disrupt particular life stages or lead to phenological mismatch within food‐webs (a terrestrial example is differential advancement of host plant and insect phenology in response to springtime warming; Uelmen et al. 2016). Throughout this text, we refer to this threshold definition as seasonally varying (SV), and to the alternative treatment as seasonally constant (SC).

Using 90th percentile SV thresholds, Burger et al. (2022) showed that combined sea surface temperature (SST)–hydrogen ion concentration (H+) events were most likely in the subtropics and occurred where the direct response of H+ to temperature overcame the competing influence of widespread negative covariance between dissolved inorganic carbon and temperature. Defining recent historical extremes based on 95th (temperature, H+) and 5th ($\Omega_{a}$) percentile SV thresholds, Hauri et al. (2024) likewise found that Gulf of Alaska surface waters were exposed to temperature and H+ extremes driven by the temperature dependence of H+. Combined temperature‐$\Omega_{a}$ events were present but less common. Wong et al. (2024) used SV 95th (temperature, H+) and 5th (oxygen) percentile thresholds, combined with an additional absolute criterion that oxygen stress events exhibit concentrations below 150 μM, in their model‐based investigation of water‐column compound extremes over the period from 1961 to 2020. They identified a 39‐fold increase in ocean volume experiencing triple extremes over that period.

Some organisms may be most sensitive to annual extreme conditions best characterized by distribution‐based thresholds defined without removing the seasonal cycle. Coral bleaching temperature thresholds, for example, are quantified in reference to the warmest part of the annual cycle (Skirving et al. 2020), with bleaching risk increasing with event intensity and duration.

The importance of accounting for adaptation, whether through phenotypic or physiological plasticity or genetic change, in response to long‐term change or previous event exposure is also becoming apparent (Bernhardt and Leslie 2013; M. C. Bitter et al. 2021; Hofmann et al. 2011). In the coral context, Logan et al. (2014) compared models of adaptation response on various time scales, showing that coral bleaching time series were broadly consistent with model‐based assessments under an adaptation framework in which the bleaching threshold was determined from a rolling 100‐year climatology. Logan et al. (2014) found that defining bleaching thresholds relative to preindustrial conditions led to significant over‐estimation of modern bleaching frequencies. Other studies have considered scenarios in which adaptation keeps pace with the long‐term trend (e.g., Burger et al. 2022).

Finally, in the context of ocean acidification stress, absolute thresholds have been investigated, such as the broadly applicable transition between super‐ and under‐saturation with respect to aragonite at $\Omega_{a}$ = 1. A less extreme threshold of $\Omega_{a}$ = 3 was empirically associated with coral habitat suitability by Guinotte et al. (2003); Kleypas et al. (1999) and further supported by Eyre et al. (2018) as an experimentally‐derived transition point between net precipitation and dissolution of coral reef sediments across multiple reef sites (specifically, $\Omega_{a}=2.92\pm 0.16$). Other organisms may respond to extremes in *p*CO_2_; for instance, citing impacts of seawater *p*CO_2_ levels between 550 and 1000 μatm on fish, McNeil and Sasse (2016) assigned a level of 1000 μatm as a threshold for environmental hypercapnia. The study identified increasing surface *p*CO_2_ seasonal cycle amplitude, driven in part by decreasing buffer capacity, as a factor driving increasing environmental hypercapnia.

Here, we diagnose projected warming (SST) and ocean acidification (H+, $\Omega_{a}$, and *p*CO_2_) extremes under a high emission and a strongly mitigating scenario. Our analysis focuses on projected changes in environmental characteristics rather than species‐specific responses. Within this context, we explore differences in projections arising from methodological choices and interpret these contrasting outcomes.

## 2 Materials and Methods

We apply a series of warming and ocean acidification extreme event definitions to historical and projected climate change scenarios and compare the implications of various thresholds, each of which may be applicable to different organisms, ecosystems, or hypotheses. We analyze the results at a global scale and at a subset of Marine Protected Areas (MPAs) and coral reef sites. Data analysis was carried out using Python version 3.9 (Van Rossum and Drake 2009) and the following libraries: SciPy version 1.10.0 (Virtanen et al. 2020), NumPy version 1.24.2 (Harris et al. 2020), pandas 1.5.3 (The pandas development team 2023; Wes McKinney 2010), Matplotlib 3.6.3 (Hunter 2007), Cartopy 0.21.1 (Met Office 2010–2015), dask 2023.2.0 (Dask Development Team 2016), xarray 2023.2.0 (Hoyer and Hamman 2017; Hoyer et al. 2023), cmocean 2.0 (Thyng et al. 2016), netcdf4 1.6.2 (Unidata 2022), GeoPandas 1.1.0 (Jordahl et al. 2020), shapely 2.0.1 (Gillies et al. 2023), and cftime 1.6.2 (Whitaker et al. 2022).

### 2.1 Model

Results are from NOAA/GFDL's Earth System Model Version 4.1 (ESM4.1, Dunne et al. 2020), for which we re‐ran segments of simulations to save additional daily diagnostic output. GFDL's Earth System Model Version 4.1 (ESM4.1) was contributed to the sixth phase of the Coupled Model Intercomparison Project (CMIP6) (Eyring et al. 2016). The model has been evaluated extensively with respect to global observations of a large suite of ocean variables including sea surface temperature (mean bias −0.07°C, RMS bias 0.68°C) (Dunne et al. 2020), pH (bias −0.00, RMSE 0.06), and *p*CO_2_ (bias −4.72 μatm, RMSE 20.45 μatm) (Stock et al. 2020). Model variability in surface ocean acidification and temperature was assessed across a range of time scales, and while the model underestimated sub‐monthly variability, correlation was found in modeled and observed multiple stressor assessments based on daily frequency data (Olson et al. 2024). Native ocean grid horizontal resolution is approximately 0.5°, with 75 vertical hybrid coordinate layers using GFDL's Modular Ocean Model Version 6 (MOM6) (Adcroft et al. 2019), while the diagnostics analyzed here are re‐gridded to fixed depth and 1° horizontal resolution. The ocean biogeochemical component is the 33‐tracer Version 2 of the Carbon, Ocean Biogeochemistry, and Lower Trophics (COBALTv2) model, with multiple plankton groups and enhanced food web dynamics (Stock et al. 2020, 2014). Carbonate chemistry uses Model of the Ocean Carbonate SYstem Version 2.0 (mocsy 2.0), (Orr and Epitalon 2015), with the Lee et al. (2010) total boron formulation, Millero (2010) carbonic acid dissolution equilibrium constants, and Dickson and Riley (1979) hydrogen fluoride dissociation constant parameterization.

### 2.2 Simulations

The variables analyzed include sea surface temperature, sea surface hydrogen ion concentration, sea surface *p*CO_2_, and sea surface aragonite saturation state. Hydrogen ion concentration (H+) is calculated from time averaged model output pH, and aragonite saturation state ($\Omega_{a}$) is calculated from time averaged model output carbonate ion concentration divided by carbonate ion concentration at aragonite saturation. The difference between H+ calculated from monthly pH and the monthly average of H+ is typically less than 1% in the open ocean but can be larger in coastal areas, particularly at high latitudes. All other variables are output directly. For a subset of CMIP6 historical and future scenarios, the last 40 years of each simulation were re‐run, saving daily average output. Forty‐year segments were analyzed in order to reduce the impact of decadal‐scale variability on the results. Thus our daily assessments of stressor conditions focus on historical simulation years 1975–2014 and future scenario years 2061–2100. Calculations extending beyond these time slices are based on monthly average fields.

#### 2.2.1 Scenarios

Rather than focusing on the more extreme SSP1‐1.9 and SSP5‐8.5 scenarios, we present and contrast results based on SSP1‐2.6 and SSP3‐7.0, which fall closer to the end of century warming range considered “more likely than not”, of 2.2°C–3.5°C (Riahi et al. 2022). Both SSP1‐1.9 and SSP1‐2.6 reflect aggressive mitigation policy, but SSP1‐1.9 restricts long‐term warming relative to preindustrial conditions to 1.5°C at 2100, whereas SSP1‐2.6 restricts warming to 2°C. Limiting warming to 1.5°C requires net zero global emissions be reached sooner, near the mid‐21st century (Riahi et al. 2022), and both goals require policy changes beyond participating nations' submitted Intended Nationally Determined Contributions at the time of the 2015 Paris Agreement (Rogelj et al. 2016). SSP5‐8.5, an estimate of “worst‐case” outcomes that has been described as “highly unlikely”, would require increasing coal use in excess of some estimates of availability (Hausfather and Peters 2020). Other pathways referenced in the following analysis are SSP2‐4.5, a medium forcing scenario associated with warming of approximately 3°C, and SSP5‐3.4‐OS, an overshoot scenario that switches from an unmitigated baseline to strong mitigation in 2040, reaching net zero emissions in 2070 (O'Neill et al. 2016; Riahi et al. 2022).

### 2.3 Threshold Definitions

We present extreme event frequencies under a suite of four quantile‐based threshold definitions as well as several additional absolute and ecosystem‐specific thresholds. We include thresholds specific to coral reefs, as an example of organism‐specific thresholds, because coral reefs are widespread in the world's oceans, ecologically and economically significant, and relatively well‐studied in the context of response to extreme conditions (e.g., Anthony et al. 2008; Drenkard et al. 2013; Kleypas and Yates 2009; Kornder et al. 2018). The four quantile‐based definitions represent permutations of SV and SC thresholds and static versus adaptive reference period. As defined in the introduction, a “seasonally varying” threshold is based on anomaly relative to the mean seasonal cycle, whereas a “seasonally constant” threshold is based on anomaly relative to the mean, with extremes therefore more likely to occur in the season associated with the minimum or maximum of the seasonal cycle. Thresholds are based on the 95th (for increasing variables) or 5th (for decreasing variables) percentile of stressor distribution, as in Hauri et al. (2024). The choice of percentile is a trade‐off between extremity (the more extreme the event, the more impactful it may be anticipated to be) and statistical robustness (closer to the tails of the distribution, the sample size is smaller); sensitivity to more or less extreme threshold choice (98th, 93rd) is explored in Figure S4. Hereafter we will refer to the 95th percentile, with the understanding that for $\Omega_{a}$, which is decreasing under climate change, we employed a 5th percentile threshold. We do not implement a minimum duration for stressor events, but we do consider one coral‐based thermal stress metric, degree heating weeks (DHW), that integrates over time with increased stress in response to longer‐lasting or repeated events within a time window. Examples of the various threshold definitions as applied to SST and $\Omega_{a}$ at one location are shown in Figure S3.

#### 2.3.1 Fixed Reference Thresholds

Fixed reference thresholds were defined based on percentile levels estimated directly from the distribution of daily model output from years 1975–2014 of the historical simulation. Thresholds were set at the 95th percentile level, so that when applied to the 1975–2014 historical simulation reference period, approximately 0.05×40×365=730 stressor days are returned. In this context, we explored both seasonally varying (SV) and seasonally constant (SC) threshold definitions, where SV thresholds shift along with the seasonal cycle, yielding a roughly constant distribution of stressor days throughout the year, and SC thresholds yield maximum stressor day frequencies in conjunction with seasonal cycle extrema.

We explored two approaches to the definition of SV thresholds. In the first, we defined a fully seasonally‐dependent 95th percentile threshold by dividing the 365‐day model year into five‐day bins and assessing the threshold level within each bin, then smoothing and interpolating back to 365‐day resolution through application of a Gaussian filter with a 10‐day timescale. In the second, after subtracting the mean seasonal cycle, we estimated a single percentile of anomaly amplitude for the whole period, irrespective of the time of year. The first approach, which accounts for seasonal differences in variance in addition to mean seasonality, leads to greater uniformity in the seasonal distribution of extreme days, while the second approach is computationally simpler and offers a more direct extension to an adaptive threshold definition based on available monthly average model outputs (see below). The two methods produce qualitatively indistinguishable results under fixed threshold analyses, and we therefore focused on the second, simpler definition; the concurrence of the two approaches is consistent with comparisons described previously (e.g., Burger et al. 2022).

#### 2.3.2 Adaptive Reference Thresholds

In addition to fixed reference period analyses, we explored shifting reference baselines, simulating adaptation. We based these calculations on a rolling climatology framework (K. Anthony et al. 2011; Logan et al. 2014; Teneva et al. 2012), in which organisms are assumed to adapt to climatological conditions over the preceding years, and the backward window length reflects an adaptation time scale. The threshold specific to a given year is calculated based on the preceding window length, N, years. We focused on analysis of the timescale *N* = 100 years, an adaptation time scale taken from the coral bleaching literature (Logan et al. 2014) but potentially applicable to a range of similarly long‐lived organisms. This time scale is used to provide an example of the impact of one representation of adaptation potential on the diagnosis of environmental extremes; some organisms may adapt on shorter or longer time scales, or not at all. Adaptation rates can depend on organism life span, population growth rates, selection strength, and pre‐existing population genetic and phenotypic variation, among other factors (Barton et al. 2020; Bernhardt and Leslie 2013; Dam 2013). Further discussion of adaptation potential can be found in the discussion (Section 4).

Complicating the adaptive analysis, only monthly data were available outside of the target time periods for all variables except temperature. Fortuitously, the annual maximum monthly mean (AMM_max_) was found empirically to be a reasonably accurate proxy for the 95th percentile level of sea surface temperature (SST), surface H+, and surface *p*CO_2_, and similarly the AMM_min_ for the 5th percentile level of surface $\Omega_{a}$ (Figure S1). In an idealized scenario with a sinusoidally varying seasonal cycle, the AMM_max_ could range from approximately the 90.5 to 95.3 percentile of the underlying distribution, with an expected value of 93.3 (see Supporting Information S1). Comparisons of RMSE and bias of AMM_max_ or AMM_min_ versus percentile in integer intervals confirmed that 93 or 94, or 6 or 7, was the nearest percentile to the AMM_max_ or AMM_min_ (Figure S2). In the seasonally constant (SC) threshold definition, we calculated the windowed mean of AMM_max_ directly as a proxy for the similarly windowed annual 95th percentile (or 18th most extreme day per year). To estimate an adaptive seasonally varying threshold, we added to the historical 1975–2014 threshold the difference in monthly mean between the rolling reference period and the historical 1975–2014 period, interpolated to the day of year.

#### 2.3.3 Absolute and Coral‐Based Thresholds

We also examined absolute (i.e., spatially and temporally constant) thresholds motivated by theoretical and/or empirical considerations as well as a degree heating week threshold empirically linked to coral bleaching event likelihood. Degree heating weeks (DHW) are calculated as defined by NOAA Coral Reef Watch (Skirving et al. 2020). Briefly, H is the difference between daily mean temperature and the Maximum Monthly Mean (MMM) for a given grid cell, where MMM is the maximum of a monthly climatology that is detrended relative to decimal year 1988.2857. Then, DHW equals the sum, over the period from 83 days prior up to a given day, of those values of H that are less than or equal to 1°C, divided by 7 (the number of days in a week). Here, the climatology used to determine MMM was based on model output from 1975 to 2014 of the historical simulation, after subtraction of the linear trend relative to 1988.2857.

We explored $\Omega_{a}$ thresholds of 1, corresponding to the transition between under‐ and super‐saturated environmental conditions, and 3, empirically linked to coral habitat extent (Kleypas et al. 1999) and more recently to an experimentally‐derived transition point between net precipitation and dissolution of coral reef sediments (specifically, $\Omega_{a}=2.92\pm 0.16$) (Eyre et al. 2018). We also applied a *p*CO_2_ threshold of 1000 μatm, a hypercapnia threshold according to McNeil and Sasse (2016). These are fairly conservative thresholds grounded in studies spanning multiple sites; individual studies in many cases report responses to less extreme conditions.

#### 2.3.4 95th Percentile Change

We consider how large the change in stressor conditions might be, based on the change in the 95th percentile of each variable's distribution from the 1975–2014 period compared to the 2061–2100 period. For these calculations, we removed the 40‐year trend over each period and thus estimated the 95th percentile of the detrended variable relative to the midpoint of the period, viz., 1995 and 2081. The seasonal cycle is not removed, and therefore changes in seasonality contribute to the reported shifts in 95th percentile levels.

#### 2.3.5 Databases Used to Identify Coral and MPA Locations

Marine protected area locations were extracted from the NOAA Marine Protected Areas (MPA) Inventory 2023–2024 (Office of National Marine Sanctuaries 2023). Grid cells containing warm‐water coral were identified based on version 4.1 of “Global distribution of warm‐water coral reefs, compiled from multiple sources including the Millennium Coral Reef Mapping Project” (UNEP‐WCMC, WorldFish Centre, WRI, TNC 2021), which includes contributions from additional sources (IMaRS‐USF 2005; IMaRS‐USF, IRD 2005; Spalding et al. 2001).

## 3 Results

### 3.1 Seasonally Constant Versus Seasonally Varying, Fixed Reference Period Thresholds

**FIGURE 1:**
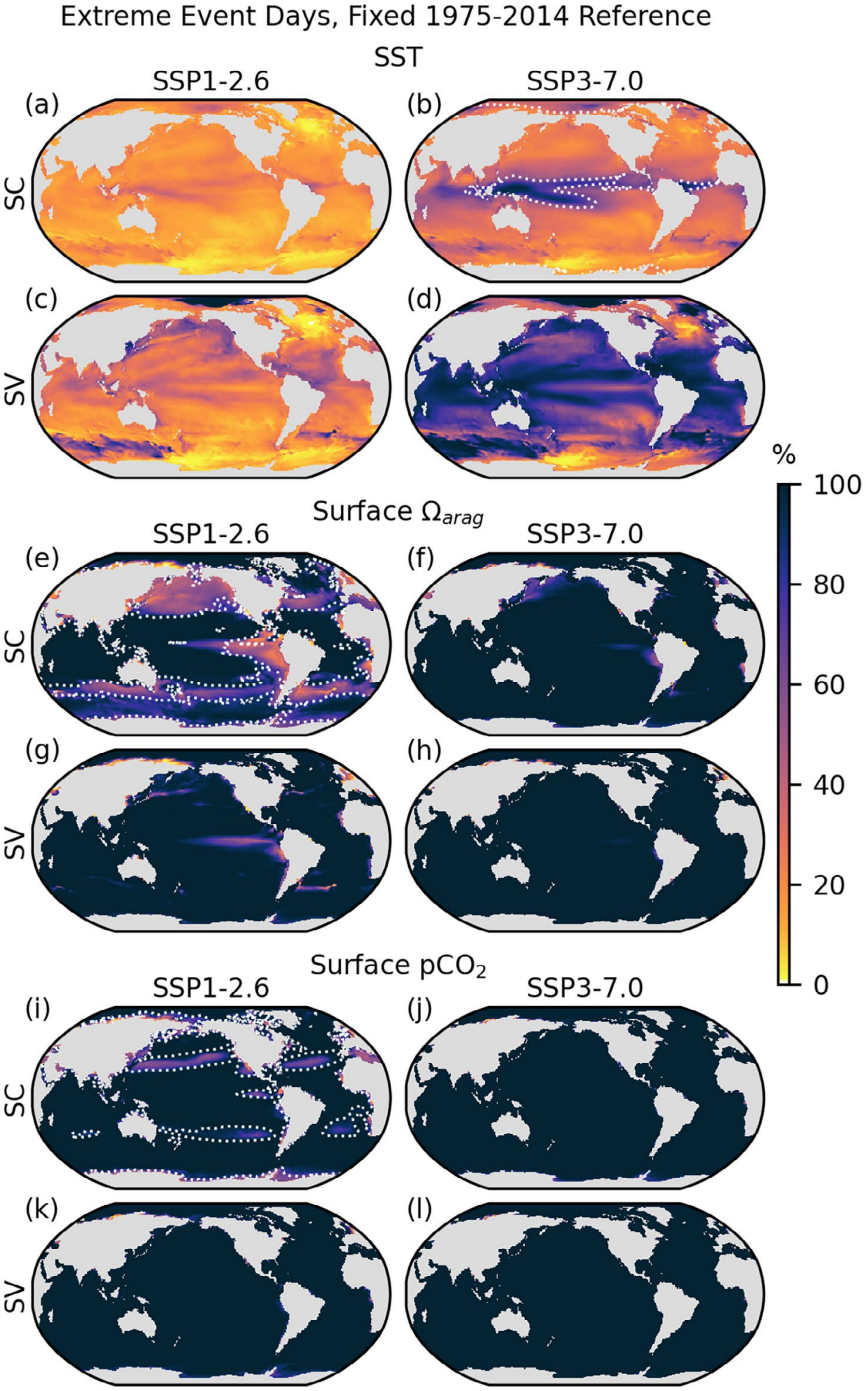
Surface 95th (SST, a–d; *p*CO_2_, i–l) or 5th ($\Omega_{a}$, e–h) percentile extreme days relative to the 1975–2014 historical reference period, according to seasonally constant (SC, a, b, e, f, i, j) and seasonally varying (SV, c, d, g, h, k, l) threshold definitions. Extreme days are calculated over the 2061–2100 period of the SSP1‐2.6 (a, c, e, g, i, k) and SSP3‐7.0 (b, d, f, h, j, l) projections and presented as a percentage of total days. The dotted white line in the SSP3‐7.0 SC threshold SST panel (b) indicates the 1°C contour of summertime seasonal cycle amplitude. The dotted white lines on the SSP1‐2.6 panels (e, i) indicate contours of annual minimum seasonal cycle amplitude of −0.15 for $\Omega_{a}$ and annual maximum seasonal cycle amplitude of 40 atm for *p*CO_2_. For differences (SV‐SC) refer to Figures S6 and S7.

First we compare assessments of extreme days over the period 2061–2100 based on SC and SV thresholds relative to the 95th percentile levels of a 1975–2014 reference period (Figure 1). The SC thresholds identify the most extreme conditions overall, whereas SV thresholds identify conditions that are unusual for the time of year. For both SST and surface ocean acidification variables (H+, $\Omega_{a}$, and surface *p*CO_2_), the regional variation in projected 2061–2100 extreme days reflects the regional pattern in threshold amplitude. Thus, these distribution‐based thresholds are primarily identifying regions of climate change susceptibility associated with patterns in historical exposure; this highlights the extent to which inferences regarding ecosystem impacts based on percentile‐derived extremes rely on an assumption that organisms are well‐adapted to local historical conditions and experience stress under conditions that were previously rare.

**FIGURE 2:**
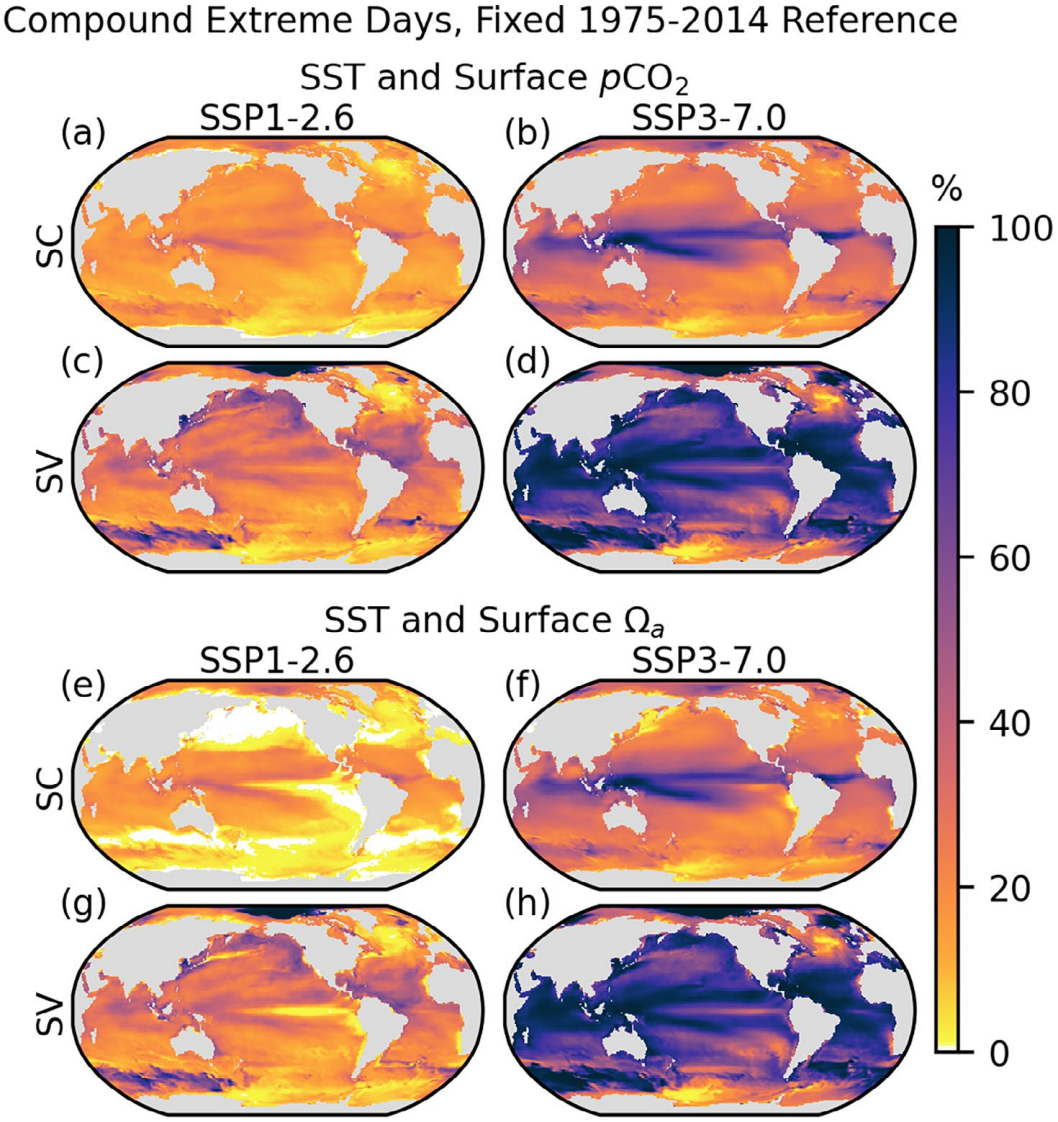
Surface compound SST with *p*CO_2_ (a–d) and SST with $\Omega_{a}$ (e–h) 95th or 5th percentile extreme days relative to the 1975–2014 historical reference period, according to seasonally constant (SC, a, b, e, f) and seasonally varying (SV, c, d, g, h) threshold definitions. Extreme days are calculated over the 2061–2100 period of the SSP1‐2.6 and SSP3‐7.0 projections and presented as a percentage of total days. White indicates zero compound days.

Dotted line contours in Figure 1 highlight the role of seasonal cycle amplitude in setting spatial patterns in extreme days under the SC threshold definition. The white dotted lines in the H+ and $\Omega_{a}$ SSP1‐2.6 SC threshold panels (e and i) surround regions of higher seasonal cycle amplitude which coincide with fewer projected extreme days. Similarly, a striking maximum in SC threshold SST extreme days, encompassing the Atlantic and Pacific inter‐tropical convergence zone, South Pacific convergence zone, and Western Pacific warm pool, coincides with a local minimum in SST seasonal cycle amplitude (the white dotted line on the SSP3‐7.0 panel (b) is the 1°C amplitude contour) under both SSP1‐2.6 and SSP3‐7.0. Cloud cover associated with consistent low pressure and convective activity during summer months in these rainy regions likely contributes to the dampening of the already small amplitude seasonal cycle in SST; the reduction in incoming shortwave radiation associated with cloud cover may moderate summertime temperatures and thus reinforce the already low amplitude seasonal variation in radiative forcing characterizing low‐latitude regions. This regional lack of historical temperature variability exposure could leave ecosystems particularly vulnerable to warming, as captured prominently by the SC threshold. Projected extremes in these areas remain frequent under the SV threshold, but stand out less relative to surrounding regions. Several of the same areas identified by Wong et al. (2024) as having frequent water‐column combined temperature‐H+ extremes over the historical period also emerge in plots of projected 2061–2100 SV combined surface SST‐*p*CO_2_ extremes—parts of the Arctic and Southern Ocean, Caribbean and Sargasso Sea, and the coastal Northwest Pacific (Figure 2).

The difference between SV and SC extreme day assessments (Figure 1) tends to be larger in extratropical regions where the seasonal cycle makes up a larger component of the overall variability. For instance, large swaths of near‐coastal area in the North Pacific stand out as regions of elevated future extremes based on SV thresholds (c and d) but not SC thresholds (a and b) for SST. Additionally, compared to SC thresholds, 2061–2100 SV threshold extreme days are more broadly geographically distributed and more numerous overall, reflecting the typically lower amplitude of the nonseasonal variability in comparison to the mid‐latitude seasonal cycle.

Large differences in 2061–2100 extreme day frequency between sea surface carbonate system variables and SST reflect the magnitude of change in these variables relative to their historical variability. Consistent with previous findings of an earlier time of emergence based on signal to noise ratio for change in ocean surface pH compared to SST (Frölicher et al. 2016), distribution‐based thresholds project near‐global $\Omega_{a}$, *p*CO_2_, and H+ extreme conditions at the end of the century even under the lower‐emissions SSP1‐2.6 scenario, whereas temperature events are regionally variable even under the higher‐emissions SSP3‐7.0 scenario. Of the ocean acidification variables, the closely related quantities *p*CO_2_ and H+ show very similar patterns in extreme days, with a slightly higher frequency at most sites that have not reached 100% extreme conditions for *p*CO_2_ compared to H+. In addition to the slightly lower overall percent of $\Omega_{a}$ extreme days compared to H+ and *p*CO_2_, the global pattern is different, with lower numbers of extreme days spread throughout the North Pacific and parts of the Southern Ocean.

Because *p*CO_2_ extreme event days are so ubiquitous under these thresholds, combined SST‐*p*CO_2_ extreme days are almost fully determined by the distribution of SST extreme days (Figure 2; compare 2a–b with 1a–b). SST‐$\Omega_{a}$ days are absent in regions where $\Omega_{a}$ extremes are less frequent under the SC threshold, reflecting the relationship of the $\Omega_{a}$ and SST seasonal cycles, driven by the tendency for higher $\Omega_{a}$ at higher temperature and anti‐correlation of DIC and SST seasonality.

### 3.2 Adaptive Thresholds

**FIGURE 3:**
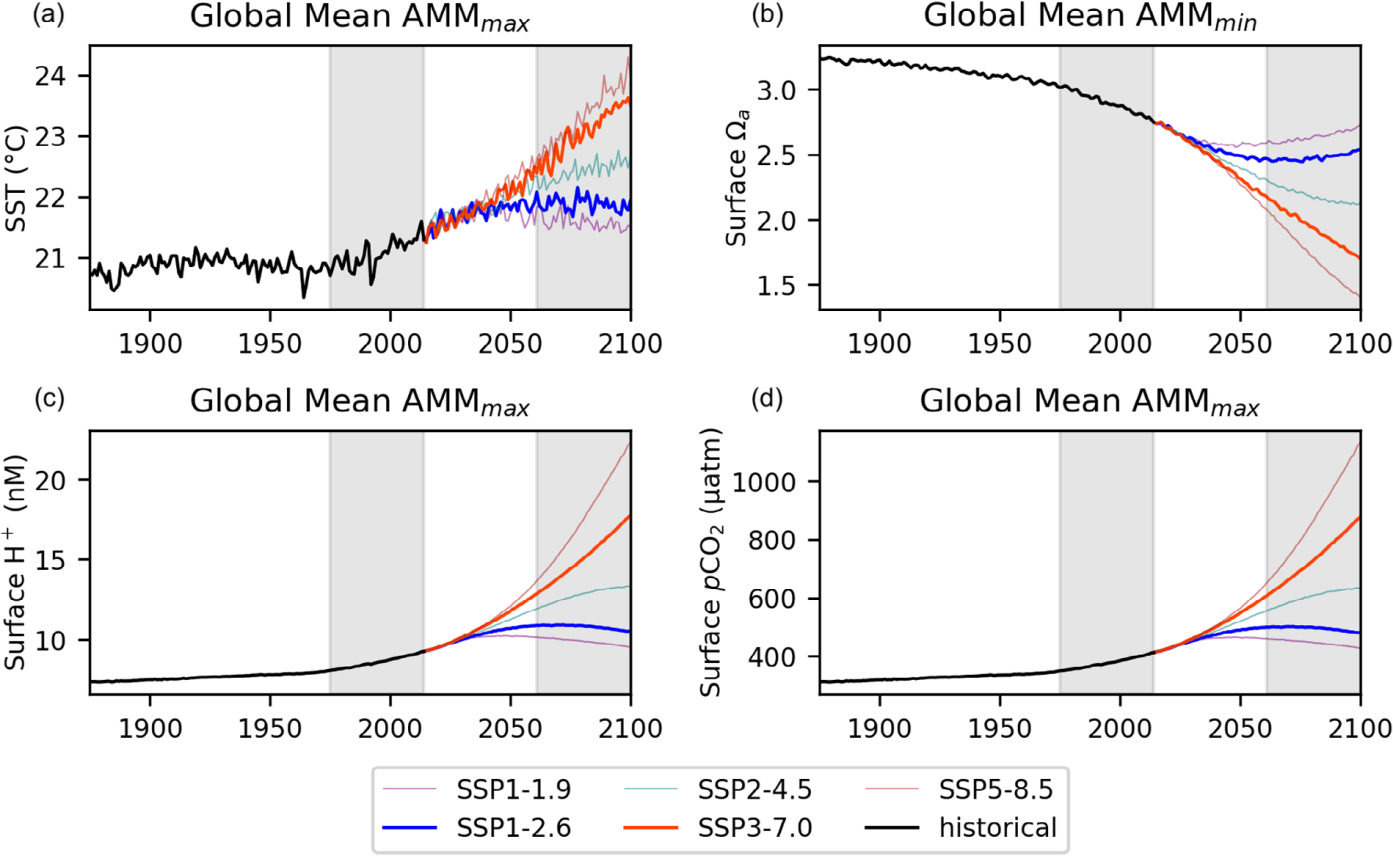
Global mean of annual extremes in monthly means maximum, AMM_max_, for (a) SST, (c) H+, and (d) *p*CO_2_, and minimum, AMM_min_, for (b) $\Omega_{a}$ based on monthly average model output, demonstrating climate change progression over time under various scenarios.

**FIGURE 4:**
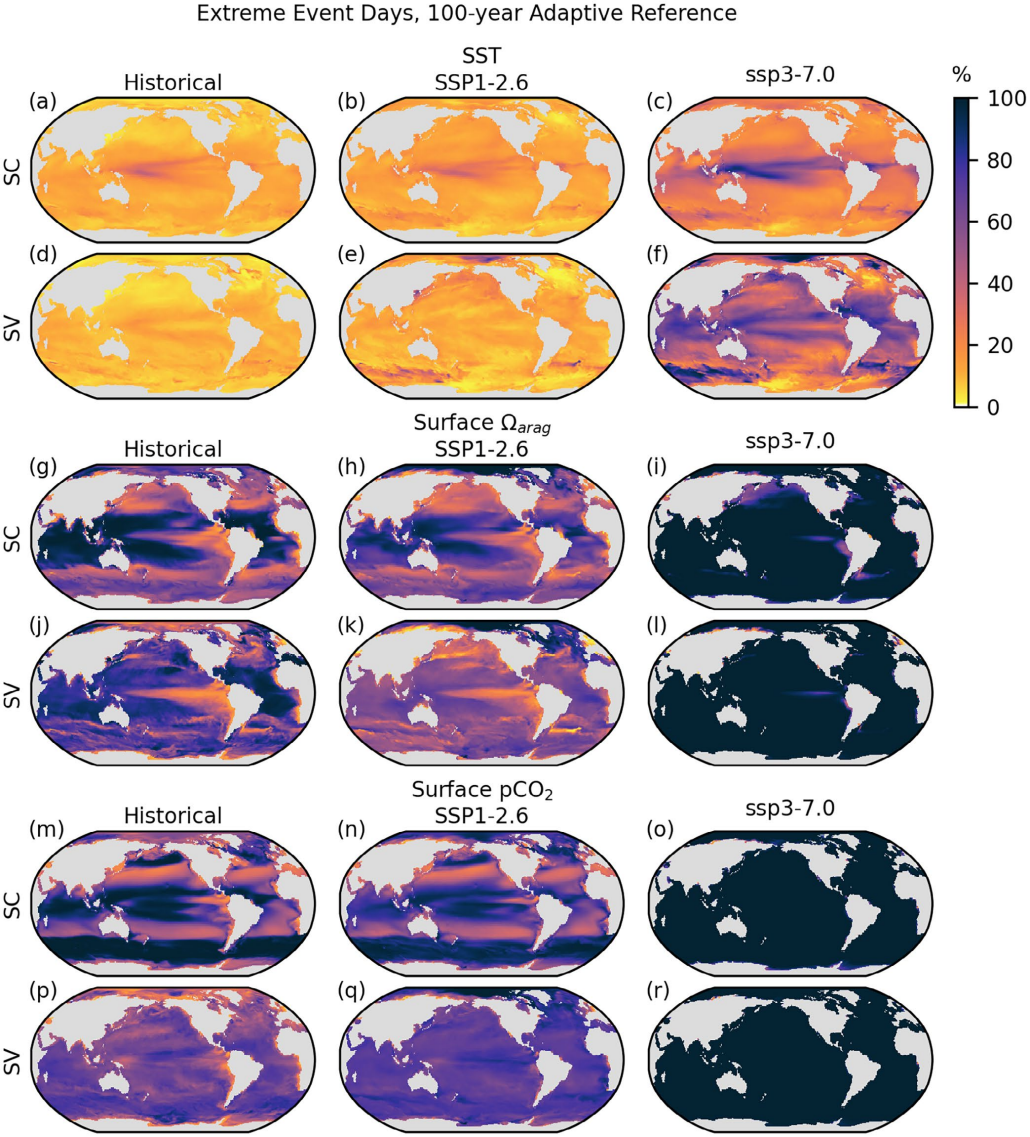
Surface ~95th (SST, a‐f; *p*CO_2_, m‐r) or ~5th ($\Omega_{a}$, g–l) percentile extreme days referenced to the preceding 100 years as described in Section 2.3, according to seasonally constant (SC, a–c, g–i, m–o) and seasonally varying (SV, d–f, j–l, p–r) threshold definitions. Extreme days are calculated over the 1975–2014 period of the historical simulation (a, d, g, j, m, p) and the 2061–2100 period of the SSP1‐2.6 (b, e, h, k, n, q) and SSP3‐7.0 (c, f, i, l, o, r) projections and presented as a percentage of total days. For differences (SV‐SC, future—historical) refer to Figures S10 and S11.

Based on the annual maximum monthly mean, we find little sensitivity of extreme values between scenarios over the next 15 years, with deviations emerging after 2040; SSP1‐2.6 keeps extremes roughly constant after that point out to 2100 while conditions in SSP5‐8.5 continue on a linear path (Figure 3). When extremes days are calculated relative to a running climatology adaptive threshold (based on the annual monthly maximum (SC) or monthly mean (SV) over the preceding 100 years—see Figure 3 and Section 2.3), thresholds on average shift slightly toward 2061–2100 conditions and therefore the total number of 2061–2100 extreme days generally decreases (Figure 4) compared to assessments under fixed reference thresholds (Figure 1). The decrease is limited under SSP3‐7.0 for $\Omega_{a}$, H+, and *p*CO_2_, however, where the threshold change is not sufficient to bring extreme days below 100% over much of the globe due that scenario's rapid rate of change in those variables relative to their variability (Figure 4). In terms of global mean change in extreme event frequency, the impact of switching from a fixed 1975–2014 reference period to a rolling 100‐year adaptive reference period under SSP1‐2.6 is strongest for SV $\Omega_{a}$ (−6182 days or 42%) and smallest for SC SST (−362 days or 2.5%). This reflects the degree to which the trend emerges from baseline variability in each context. Results based on a shorter, 50‐year reference period consistent with more rapid adaptation are included in Figure S9 and exhibit further lessening of the number of extreme days, particularly under SSP1‐2.6.

**FIGURE 5:**
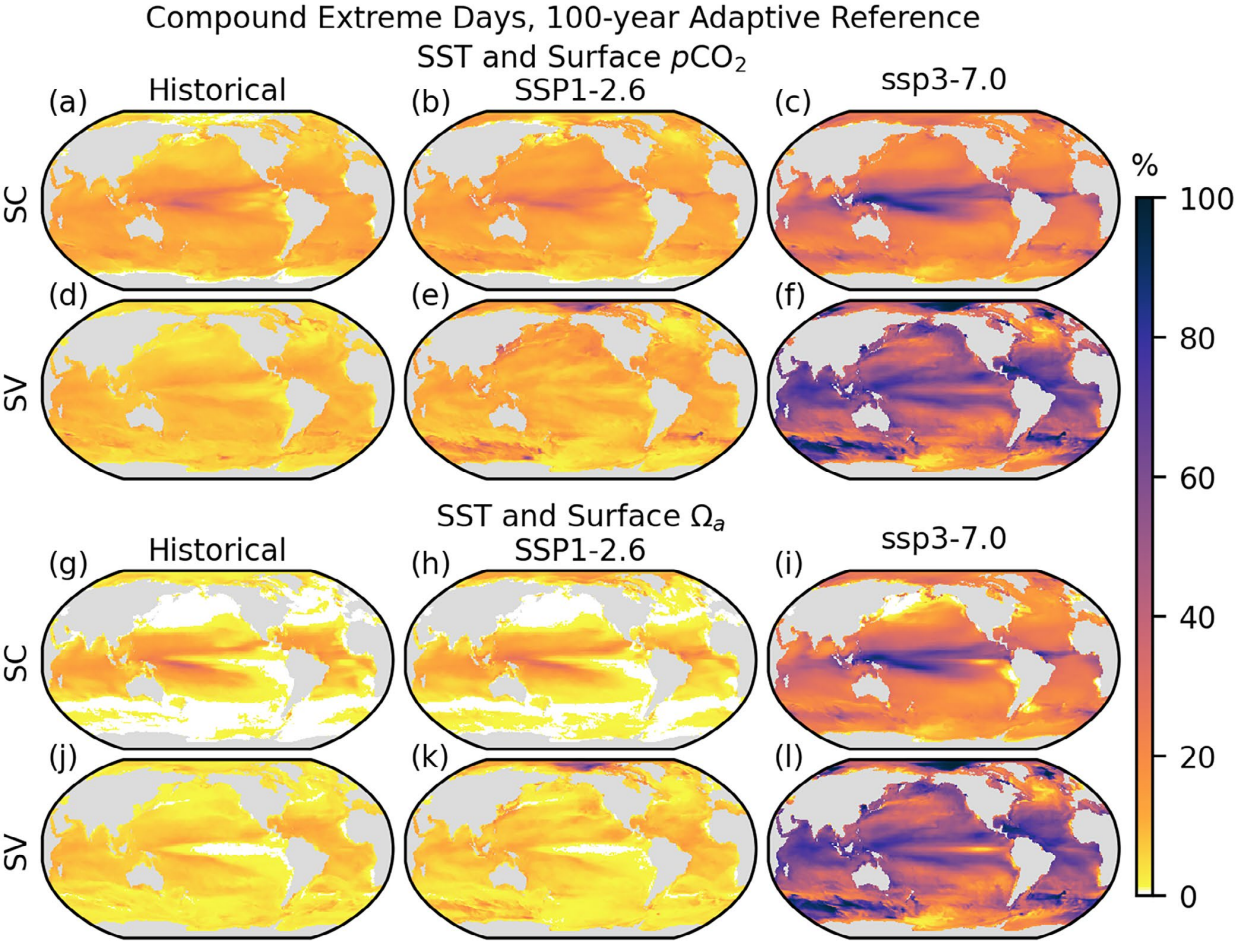
Surface compound SST with *p*CO_2_ (a–f) and SST with $\Omega_{a}$ (g–l) ~95th or ~5th percentile extreme days referenced to the preceding 100 years as described in Section 2.3, according to seasonally constant (SC, a–c, g–i) and seasonally varying (SV, d–f, j–l) threshold definitions. Extreme days are calculated over the 1975–2014 period of the historical simulation (a, d, g, j) and the 2061–2100 period of the SSP1‐2.6 (b, e, h, k) and SSP3‐7.0 (c, f, i, l) projections and presented as a percentage of total days. White indicates zero compound extreme days.

Applied to the historical period, thresholds shift toward an earlier reference, increasing the total number of extreme days from the 5% prescribed by the fixed reference threshold definition. Defined in reference to rolling antecedent windows, the adaptive threshold method produces commensurate assessments across time periods. In reference to the preceding 100 years, SSP1‐2.6 extreme days are similar in number to the 1975–2014 historical period (Figures 4 and 5). In contrast, under SSP3‐7.0, 2061–2100 extreme days are nearly as pervasive in the adaptive context as when diagnosed relative to the fixed historical framework due to continued rapid change through most of the 21st century. Thus, in the adaptive framework, striking differences emerge between SSP1‐2.6 and SSP3‐7.0 reflecting differing stressor trajectories (Figure 3). Globally, there is an acceleration of warming and ocean acidification under SSP3‐7.0 and a deceleration and ultimate stabilization in the SSP1‐2.6 scenario. The extent of mitigation is such that under SSP1‐2.6, the number of extreme days relative to a shifting baseline is actually slightly less in 2061–2100 compared to 1975–2014 for aragonite saturation state over much of the globe, excluding the Arctic (Figure 4). This reflects the pattern in global surface aragonite saturation state over time, with minimum global mean AMM_min_ occurring early in the 2061–2100 period under SSP1‐2.6, ahead of maximum global mean AMM_max_ for SST.

### 3.3 Absolute and Coral‐Specific Thresholds

In addition to deviation from past typical conditions, ecosystems impacts may be triggered when specific biological or chemical thresholds are reached. These may be governed by chemical equilibria (saturation state), metabolic, or other factors and may be general or specific to a particular group of organisms. We consider two relatively severe absolute thresholds of broad applicability to marine animals ($\Omega_{a}$ < 1 and *p*CO_2_ > 1000 μatm; McNeil and Sasse 2016); a coral‐specific absolute threshold of $\Omega_{a}$ < 3 empirically related to coral habitat extent (Guinotte et al. 2003; Kleypas et al. 1999) and a transition to reef dissolution (Eyre et al. 2018); and a more complex thermal threshold of 4° heating weeks (DHW) that has been associated with coral bleaching (Eakin et al. 2009; Skirving et al. 2020).

**FIGURE 6:**
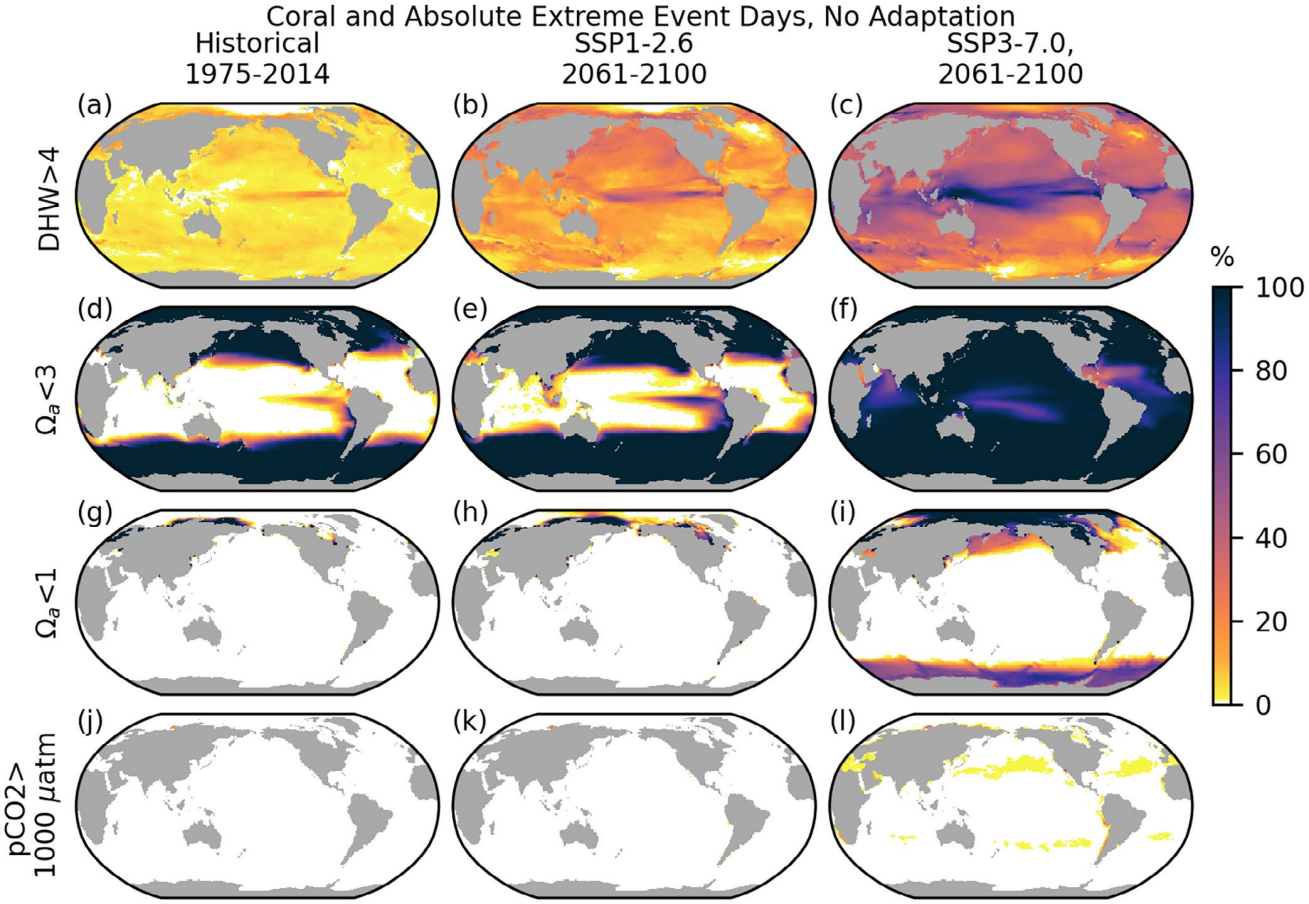
Extreme event days under absolute and warm‐water coral thresholds, assuming no adaptation. All thresholds are shown for the entire globe, but warm‐water corals are found primarily between 30 N and 30 S. Coral‐containing grid cells are highlighted in Figure 7. Rows, top to bottom: Surface thermal conditions exceeding 4° heating weeks (coral, a–c); surface $\Omega_{a}$ < 3 (coral/general, d–f); $\Omega_{a}$ < 1 (general, g–i); *p*CO_2_ > 1000 μatm (fish, j–l). White indicates zero event days.

The more severe threshold of $\Omega_{a}$ < 1 is increasingly exceeded in the surface waters of the Canadian Arctic under SSP1‐2.6 compared to the historical period (Figure 6g,h). Consistent with previous findings (Steinacher et al. 2009; Terhaar et al. 2021), surface aragonite undersaturation under SSP3‐7.0 (Figure 6i) is pervasive over the 2061–2100 period throughout the Arctic, where dilution of alkalinity due to freshening compounds $\Omega_{a}$ decline due to carbon uptake. Undersaturation occurs up to 20%–40% of the time in the northern North Pacific and northwestern North Atlantic, as well as at a high frequency in the Southern Ocean. Eastern ocean margins are hot‐spots for surface hypercapnia under SSP‐3.70 (Figure 6l), with additional affected areas in semi‐enclosed coastal seas, parts of the Russian Arctic, and parts of the subtropical gyres, particularly in the North Atlantic and North Pacific. However, model residence times, an indicator of the degree of water mass isolation or exchange that can correlate with influence of local biogeochemical activity on tracer concentrations, can be error‐prone in coastal shelf environments, particularly at lower resolutions of 1 or 1/2° (Liu et al. 2019) as in ESM4.1, leading to greater uncertainty there. The environmental hypercapnia threshold of *p*CO_2_=1000 μatm is exceeded for 105 days (0.7% of the 2061–2100 period) in a small section of the subtropical Atlantic and exceeded with lower frequency in parts of the other four subtropical gyres under SSP3‐7.0.

**FIGURE 7:**
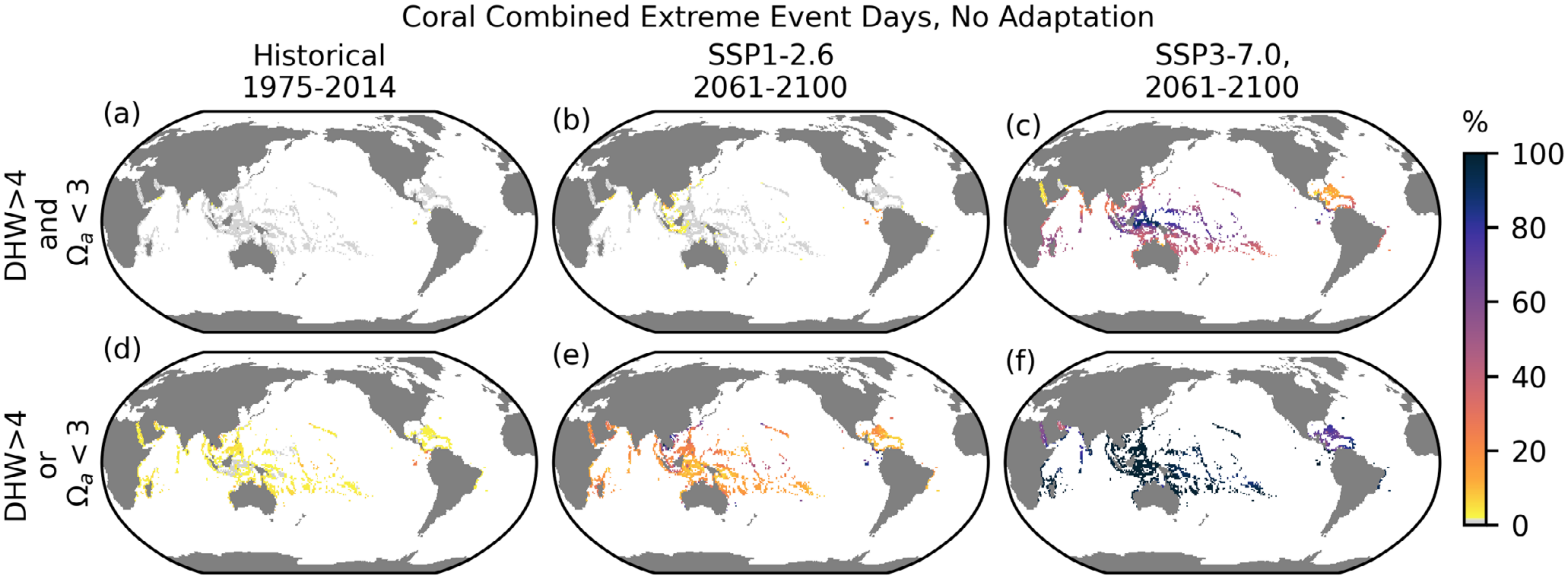
Percentage of days that are surface compound extreme event days based on thresholds of > 4° heating weeks (DHW) and $\Omega_{a}$ < 3 at grid cells containing coral reefs. Upper (a–c): Compound event days with simultaneous DHW > 4 and $\Omega_{a}$ < 3 (intersection). Lower (d–f): Days exceeding either or both conditions (union). White indicates grid cells without coral reefs and light gray indicates coral reef grid cells with zero extreme days.

Under SSP1‐2.6, 2061–2100 sees constriction in coral habitat relative to 1975–2014 based on the $\Omega_{a}$ < 3 threshold, with significant overlap between emerging $\Omega_{a}$ extremes and warm‐water coral reef sites in the vicinity of Indonesia and the Indochinese Peninsula (Figures 6d,e and 7a,b). Under SSP3‐7.0, the spatial extent of $\Omega_{a}$ < 3 occurrence is near‐global; only 28 grid cells (< 7000 km^2^) do not experience one or more days with $\Omega_{a}$ < 3 over the 2061–2100 period.

The DHW coral bleaching metric (Figure 6a–c) differs from the strictly percentile‐based SST threshold used in the previous sections in that extreme classification is initiated at 1°C above the MMM, and the DHW integrates over time, weighting event severity by amplitude and by duration up to 12 weeks. Compared to a strictly percentile‐based SC threshold (Figure 1a,b), a degree heating week threshold shows broadly similar spatial patterns, with the greatest number of extreme days close to the equator (Figure 6b,c). The convergence zones stand out in SSP3‐7.0 (Figure 6c), but not in SSP1‐2.6 (Figure 6b) in this non‐adaptive DHW framework. Additionally, where a reduction in event frequency might be anticipated due to the higher threshold of 1°C over the MMM, the overall number of extreme days increases in some locations, such as the eastern Equatorial Pacific and to a lesser extent in much of the North Pacific and eastern North Atlantic under SSP1‐2.6, and much of the globe under SSP3‐7.0 (Figure 6a,b compared to Figure 1b,c). These differences are consistent with the cumulative nature of the DHW framework, which assigns longer‐lasting (and larger amplitude) events greater severity and can extend event duration forward in time from a large amplitude or long‐lasting warm anomaly.

Combined effects of multiple stressors may be anticipated to affect ecosystems more intensely. We present combined extreme frequencies at grid locations known to contain coral, noting that corals occurring deeper in the water column may experience higher frequency of $\Omega_{a}$ < 3 conditions and could also be under‐represented by the satellite‐informed reef‐location product employed in this analysis. Days simultaneously surpassing DHW > 4 and $\Omega_{a}$ < 3 in surface waters at coral sites are extremely rare over the historical period (Figure 7a–c). However, in 2061–2100 under SSP1‐2.6, the frequency increases over those sites and low‐frequency events spread to a wider area, affecting the southern Arabian Peninsula, Indochinese Peninsula, Indonesia, as well as Panama and the equatorial Eastern Pacific. Under SSP3‐7.0, simultaneous events are widespread, with near‐100% frequency at many coral sites near the equator, particularly in the western Pacific warm pool east of Indonesia, and significant frequencies in the Indian Ocean. DHW and $\Omega_{a}$ event days tend not to co‐occur, and therefore, if we consider the total number of days experiencing either type of event (Figure 7d–f), the affected duration increases, such that under SSP3‐7.0, a majority of coral locations experience at least one type of extreme nearly all of the time.

### 3.4 Adaptation Burden/Extinction Versus Extirpation

We have explored variability‐based assessments that tend to emphasize susceptibility associated with low historical variability, and absolute thresholds reflecting instantaneous conditions. Next, we consider how large the change in extreme conditions is projected to be, based on the change in the 95th percentile of each variable's distribution from the 1975–2014 period to the 2061–2100 period (Figure S12). This percentile includes the variability due to the seasonal cycle and therefore roughly corresponds to the SC thresholds applied previously; however, the 40‐year linear trend over each period has been removed. We interpret the shift in this 95th percentile level as indicative of the change to which an organism responsive to 95th percentile extremes (roughly 18th hottest or most acidic day of the year) would have to adapt so that a 2081 95th percentile event would yield the same response as a 1995 95th percentile event (Figure S12).

The change in 95th percentile SST is similar to the change in mean SST under SSP1‐2.6 and SSP3‐7.0, but is amplified over large swaths of the Northern Hemisphere (Figures S12 and S13). This amplification is largely due to increases in seasonal cycle amplitude previously reported by Kwiatkowski et al. (2020); in fact, the regions of amplification in the North Pacific and North Atlantic are absent from plots of the deseasonalized 95th percentiles (not shown). The ESM4.1 change in mean SST, in turn, is consistent with the CMIP6 multimodel mean reported by Kwiatkowski et al. (2020), despite the slightly different time periods they analyzed (1995–2014 and 2080–2099). Of the stressors analyzed, SST change shows the greatest spatial variability (Figure S12). The simulations exhibit slight cooling in the northwest Atlantic, consistent with the North Atlantic warming hole (e.g., Keil et al. 2020); however cooling at the 95th percentile level is less than cooling of the mean. While SST change at the 95th percentile level is variable and does not always exceed historical variability, change in the carbonate chemistry variables ($\Omega_{a}$, H+, and *p*CO_2_) is more globally uniform and larger relative to historical variability. After temperature, the variable showing the greatest level of amplification of change at the 95th percentile level relative to the mean is surface *p*CO_2_, which shows amplification in subtropical gyres, particularly in the northern hemisphere.

**FIGURE 8:**
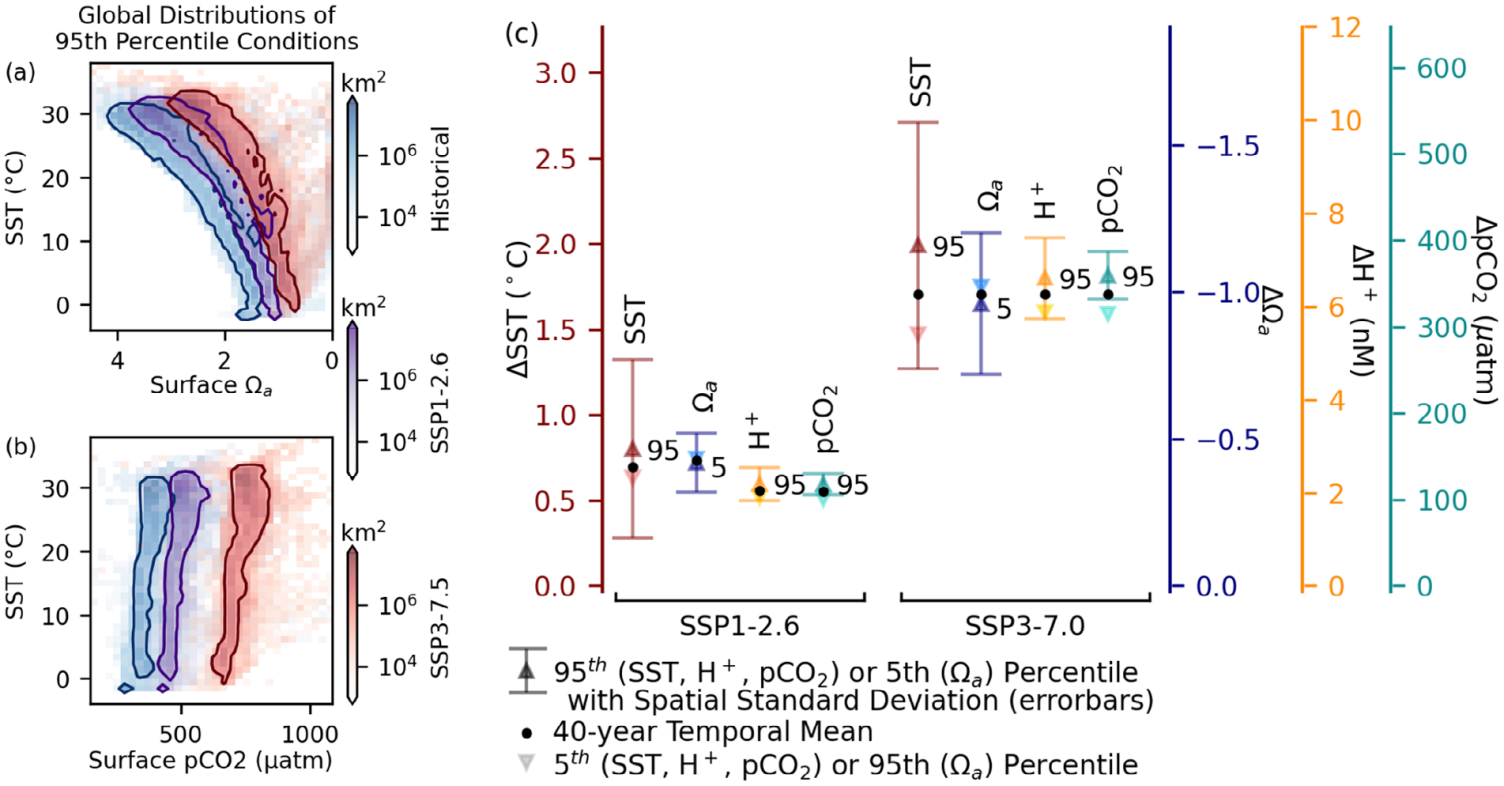
Changing distributions. Left: 2‐d histograms showing the distributions of (a) 95th percentile SST and 5th percentile surface $\Omega_{a}$ conditions and (b) 95th percentile SST and surface *p*CO_2_. The solid contours enclose conditions representative of approximately 90% of surface ocean grid cells. Right: (c) 1995–2081 global mean change in 95th percentile, 40‐year mean, and 5th percentile. Change in $\Omega_{a}$ is presented with axis direction reversed so that increasing stress is oriented up the page, as for the other stressors. Errorbars represent the spatial standard deviation of the change in 95th (SST, H+, *p*CO_2_) or 5th ($\Omega_{a}$) percentile.

Figure 8a,b shows the extent to which future global surface 95th percentile carbonate chemistry and temperature conditions separate from historical conditions under SSP1‐2.6 and SSP3‐7.0, and illustrates two aspects of combined temperature‐acidification change. First, when considering either $\Omega_{a}$ or *p*CO_2_, the small degree of overlap between present and future distributions, particularly in the high CO_2_ pathway (SSP3‐7.0), indicates that the combinations of warming and carbonate chemistry extremes experienced in the 2061–2100 period will largely be globally unprecedented in the historical 1975–2014 context, even at many sites where neither the warming or acidification would be similarly unprecedented on its own. The second point is that the potential for mitigation through range migration may be extremely limited if both OA and thermal extremes are factors. In Figure 8a,b, both OA variables' axes are oriented so acidification proceeds to the right (decreasing $\Omega_{a}$ and increasing *p*CO_2_), and the bounded regions represent 90% of global ocean surface area. Without adaptation, an organism at the edge of its warming or acidification tolerance during the historical period could therefore be limited in migration, in the absence of adaptation, to ocean area between that (SST, OA) point and the *x*‐ or *y*‐axis. A move to a future area with the historical temperature maximum would in many cases be a move to lower aragonite saturation state, potentially trading one stressor for another. The different orientation of the populated regions in SST‐surface *p*CO_2_ space compared to SST‐$\Omega_{a}$ reflects differences in correlation, but there is even greater separation between the historical and future regions along the *p*CO_2_ axis. For comparison, the corresponding figure based on the distribution of mean values rather than 95th or 5th percentile is included (Figure S14).

### 3.5 Local Stressor Frequency Projections: MPA Sites

**FIGURE 9:**
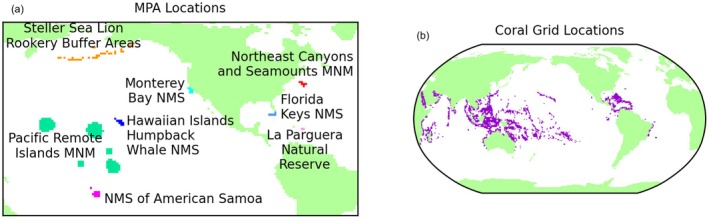
(a) Model grid‐located selected marine protected areas and (b) grid cells containing known warm‐water coral reefs.

**FIGURE 10:**
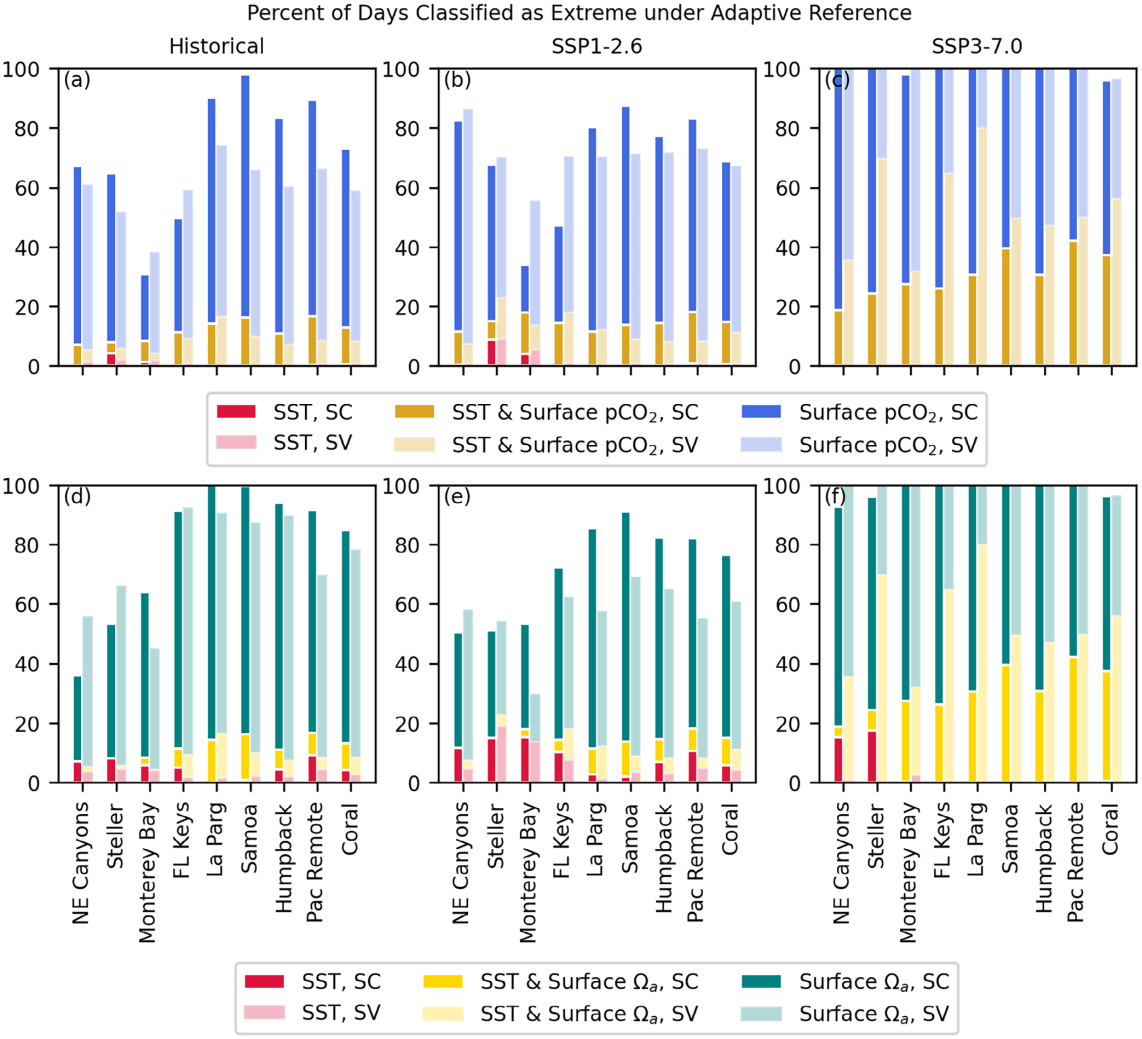
Stacked bar charts showing percent of days area that are extreme SST and/or *p*CO_2_ days (a–c) or SST and $\Omega_{a}$ days (d–f) across scenarios at U.S. federally protected sites with an adaptive reference period. Columns, left to right, are historical 1975–2014 (a, d), SSP1‐2.52061–2100 (b, e), and SSP3‐7.02061–2100 (c, f). SC, seasonally constant thresholds; SV, seasonally varying thresholds.

**FIGURE 11:**
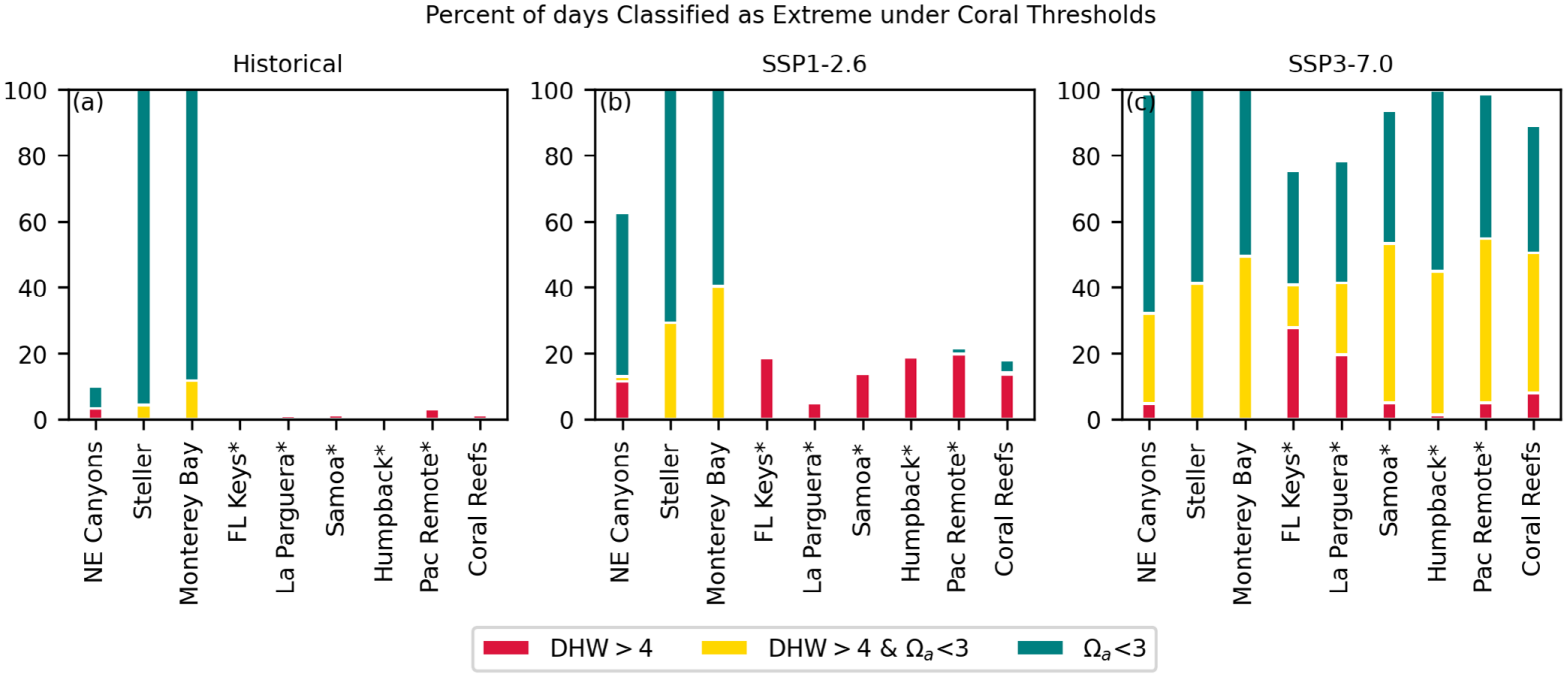
Stacked bar charts showing percent of days area that are extreme according to fixed coral thresholds. Columns, left to right, are historical 1975–2014 (a), SSP1‐2.52061‐2100 (b), and SSP3‐7.02061‐2100 (c). Asterisk indicates MPA sites with coral reefs.

Site‐specific assessments at MPA locations illustrate regional and inter‐scenario differences in stressor frequency (Figures 9, 10, 11). While some variability is present between sites, the most striking differences are between scenarios, with nearly complete transition to H+ and $\Omega_{a}$ stress conditions in the higher emissions scenario, even in the adaptive framework (Figures 10 and 11). The degree of overlap between temperature and H+ or $\Omega_{a}$ stress varies between sites, with less stress overlap at Steller and Monterey Bay than elsewhere. In contrast, warming stress days are almost always also H+ or $\Omega_{a}$ stress days at many tropical sites, including La Parguera and American Samoa, in terms of variability‐based thresholds (SC and SV). In terms of an absolute $\Omega_{a}$ threshold of 3, however, these tropical sites are among the least frequently stressed (Figure 11). Thus, while $\Omega_{a}$ stressor days are common at tropical sites when diagnosed relative to historical variability in both scenarios, the mitigation scenario avoids crossing the $\Omega_{a}$ < 3 threshold. However, thermal stress is still projected for many coral sites based on a threshold of four degree heating weeks.

Of the sites analyzed, Monterey Bay, NE Canyons and Steller are among those less impacted in terms of frequency of percentile‐based extreme events over the 2061–2100 period under SSP1‐2.6 (Figure 10 and Figure S15). In contrast, these sites are among the most impacted by aragonite undersaturation (Figure 11). The lower percentile‐based event frequencies are derived from variability and low aragonite saturation over the historical period; all three sites had already begun to experience $\Omega_{a}$ < 3 extreme days over 1975–2014 (Figure 11 and Figure S16).

## 4 Discussion

In Section 3.1, we showed that of the three acidification‐related variables considered, *p*CO_2_ exhibits the highest sensitivity to climate change in terms of days exceeding a 95th percentile theshold based on a fixed historical reference period, followed closely by H+, which has very similar global patterns (Figure 1). Additionally, *p*CO_2_ showed the largest increases in its 95th percentile relative to the increase in its mean (Figure 8 and Figure S13). This is consistent with previous studies that have identified increasing seasonal cycle amplitude, as well as an increasing mean state, in *p*CO_2_ (McNeil and Sasse 2016). Aragonite saturation state is the most moderate but still exhibits constant exceedence of fixed historical 95th percentile thresholds over much of the globe under both future scenarios.

For warming and to an even greater extent for H+, $\Omega_{a}$, and *p*CO_2_, regional differences in historical variability dominate regional patterns in variability‐based stressor frequency projections (see Figures 1 and 4). Thus, spatial patterns in historical variability can inform understanding of method‐based differences in stressor frequency projections. For instance, in some equatorial regions (e.g., the convergence zones), the DHW threshold projects fewer equatorial stress days than the 95th percentile SC SST threshold because the 1°C threshold offset included in the DHW definition represents a larger proportional change in threshold amplitude where variability is low. In other regions (e.g., the eastern equatorial Pacific), the timescale and magnitude of the non‐seasonal variability can lead to more DHW threshold extreme days than 95th percentile SC SST extreme days.

Projected H+, $\Omega_{a}$, and *p*CO_2_ changes are large compared to their spatial and temporal variability. However, model variability in these quantities is also likely underestimated. While GFDL‐ESM4.1 accurately represents SST variability and surface air *p*CO_2_ variability across a range of time scales, the oceanic variability observed in *p*CO_2_ and pH at surface moorings is under‐represented, particularly in coastal regions or on timescales of less than a month (Olson et al. 2024). This is consistent with more severe observation‐based compared to model‐based predictions of hypercapnia by McNeil and Sasse (2016), notwithstanding their assessment of potential methodological bias associated with steady‐state assumptions. At model grid locations representing coastal and reef sites, the footprint of a 100 km^2^ grid cell can encompass a heterogenous range of fine‐scale environments, and modeled conditions are anticipated to be representative of the open‐ocean end of that range. Missing local processes may modify temperature and OA variables, likely amplifying real‐world extremes beyond what is seen in the model. For example, just as McNeil and Sasse (2016) identified increased *p*CO_2_ seasonal cycle amplitude due to decreased buffer capacity, Shaw et al. (2013) demonstrated increased *p*CO_2_ amplitude due to reduced buffer capacity in the context of diurnal variability associated with high biological productivity in shallow coastal environments such as coral reefs.

In both the fixed and adaptive reference context, where $\Omega_{a}$ extreme days are rare, they tend not to co‐occur with temperature stress days, leading to regions without compound SST‐$\Omega_{a}$ days (Figures 2 and 5). This is most often the case in the SC thresholds, where extreme days are focused at the peak or nadir of the seasonal cycle. Observed open‐ocean surface $\Omega_{a}$ is higher in warmer months than colder months (Jiang et al. 2015; Takahashi et al. 2014). Temperature dependence of $\Omega_{a}$ in the surface ocean can arise from three direct mechanisms promoting anticorrelation of $\Omega_{a}$ and SST: the shift in inorganic carbon equilibrium to increased carbonate ion concentration at higher temperature, the decrease in the apparent solubility product for aragonite with higher temperature, and the greater solubility of carbon, favoring gas flux into the ocean and thus increased DIC and $\Omega_{a}$, at lower temperature (Jiang et al. 2015). An additional indirect factor is increased stratification at higher surface temperatures, reducing mixing of high DIC waters from below; Burger and Frölicher (2023) identified increased mixing of high DIC surface waters to be a primary driver of low $\Omega_{a}$ events.

When severity is viewed in an adaptive context, relative to a 100‐year shifting baseline, the mitigation associated with SSP1‐2.6 emerges as highly impactful. This time scale will be relevant for some species but not others; species may adapt over shorter or longer time periods, or not at all. The present analysis sheds light on environmental factors known to influence adaptation potential, reflecting the rate at which climate change proceeds under different scenarios and at different locations relative to a shifting reference period. However, there are many other biophysical, genetic, and ecological factors that contribute to adaptation potential.

Both rapid adaptation through phenotypic or physiological plasticity over one or multiple generations and trait evolution requiring many generations can contribute to organismal adaptation to environmental change (Barton et al. 2020; Bernhardt and Leslie 2013; Hofmann and Todgham 2010; Stillman et al. 2015). In the context of phenotypic plasticity, species in high variability environments may have greater capacity for adaptive response, yet may also be already existing near an upper tolerance limit (Bernhardt and Leslie 2013; Kroeker et al. 2020). Additionally, species may be better able to match their phenotype to a changing environment when those changes are predictable (Bitter et al. 2021; Kroeker et al. 2020).

In the laboratory, adaptation responses are more easily studied in organisms with shorter generation times. The potential for genetic adaptation in response to both warming and acidification has been demonstrated experimentally in marine microbes such as unicellular phytoplankton in experiments spanning hundreds of generations (Bernhardt and Leslie 2013; Sunday et al. 2014). Fitness‐based and genetic evidence of adaptation of a zooplankton species to simultaneous warming and acidification was detected after only the third generation in a study of the zooplankton species *Acartia tonsa* in a study lasting 25 generations, or 1 year, but recovery was more limited under combined warming and acidification than under warming alone (Dam et al. 2021). A 500‐generation study of the calcifying coccolithophore *Emiliana huxleyi* showed an initial adaptive response somewhat mitigating the effects of increased CO_2_ (Lohbeck et al. 2012); however, a longer 2100‐generation (4 year) study found a subsequent reversion of phenotypic responses including calcification (Schlüter et al. 2016). Based on a combination of a factorial breeding study with evolutionary modeling, Sunday et al. (2011) estimated a 50‐year adaptation response to future CO_2_ conditions in a species of sea urchin, but a slower response of a less phenotypically and genetically variable mussel species. Conover et al. (2006) reviewed evidence of adaptive divergence through selection on contemporary timescales, from tens to 200 years.

The ability of evolutionary adaptation to keep pace with environmental change depends on many factors, increasing with maximum population growth rate, strength of selection, existing population genetic and phenotypic variance, and heritability but decreasing with generation time and with the ability of plastic responses to keep pace with environmental change, reducing selection pressure (Bernhardt and Leslie 2013; Bitter et al. 2019; Dam 2013). Additionally, in contrast to response to continuous change, adaptation to intermittent stress conditions can be inhibited by fitness penalty during intervals between stress episodes (Dam 2013). Even where adaptation improves fitness in the presence of a stressor, it may be associated with changes in organismal function (e.g., photochemical performance, Zhong et al. 2021). Thus, mitigation of climate change impacts through acclimation and adaptation may be expected to vary widely among species and across ecosystems and may not buffer ecosystems completely against the impacts of change. Furthermore, adaptive potential may be limited by physical and chemical constraints; for example, the $\Omega_{a}$ < 3 threshold estimated by Eyre et al. (2018) as a transition from net reef accretion to dissolution reflects a balance between biotic (production) and abiotic (dissolution and physical loss) processes.

If species are unable to adapt sufficiently rapidly to changing conditions, another potential mode for species survival is migration to a new habitat range. However, as shown, organisms susceptible to stress due to both warming and acidification extremes may have significantly reduced options, as in many cases moving to an area more suitable with respect to temperature extremes will entail worse aragonite saturation state, and vice versa, the context of both the mean state and extreme events. For *p*CO_2_ (or H+) and temperature, the issue is not as much a trade‐off between the two as that the future surface *p*CO_2_ extreme conditions depart so strongly from the historical that no part of the surface ocean could offer a reprieve. In the event that species are able to shift their range to more hospitable conditions, shuffling of individual habitat ranges could lead to food chain disruptions. The lack of overlap between historical and future combined temperature‐OA extreme conditions suggests that extinction, rather than only local extirpation, is a risk for organisms unable to sufficiently adapt.

Of the MPA sites considered, all exhibited projected extreme events over the 2061–2100 period under both scenarios. Thus, interdisciplinary ecosystem monitoring, including incorporation of evolutionary processes (Cocciardi et al. 2024; Hoffmann and Sgrò 2011) is called for at all sites to facilitate early detection of impacts and inform management decisions. The differing projections under various threshold definitions reflect varying potential modes of impact and susceptibility, and understanding of real‐world impacts could be gained through observations and comparisons across sites. Much could be learned from studying and comparing ecosystem responses across the range of MPA sites in relation to varied extreme event metrics.

Our results highlight differences in patterns of regional extreme event projections resulting from the choice of SC or SV thresholds as well as potential for maintenance of near‐historical levels given a combination of mitigation and ecosystem adaptation. However, the level of adaptation required is may be more likely for organisms with shorter life spans, and will be influenced by a variety of ecological, physiological, and genetic factors. The low‐CO_2_, mitigation scenario combined with adaptation could lead some organisms to experience impacts from extreme conditions only slightly more severe than those experienced over the historical period. Avoidance of severe ecosystem impacts may require both human‐led mitigation and natural adaptation/resiliency.

## Author Contributions

**Elise M. Olson:** formal analysis, investigation, methodology, software, validation, visualization, writing – original draft. **Jasmin G. John:** conceptualization, funding acquisition, investigation, project administration, resources, supervision, writing – review and editing. **John P. Dunne:** conceptualization, funding acquisition, investigation, project administration, resources, supervision, writing – review and editing. **Charles A. Stock:** conceptualization, funding acquisition, investigation, writing – review and editing. **Elizabeth J. Drenkard:** conceptualization, funding acquisition, investigation, writing – review and editing.

## Conflicts of Interest

The authors declare no conflicts of interest.

## Supporting information


Data S1.

## Data Availability

Data supporting the findings of this study are openly available from the NOAA National Centers for Environmental Information at https://doi.org/10.25921/776n‐5w58. Analysis code supporting this study is available through GitHub at https://github.com/e‐olson/extremes‐OAW‐GCBmanuscript‐code and archived with Zenodo at https://doi.org/10.5281/zenodo.15832935. CMIP6 data were obtained from the Earth System Grid Federation at https://esgf‐data.dkrz.de/search/cmip6‐dkrz/, with DOIs https://doi.org/10.22033/ESGF/CMIP6.1414 and https://doi.org/10.22033/ESGF/CMIP6.8597. Geospatial data identifying marine protected areas were obtained from the Office of National Marine Sanctuaries at https://www.fisheries.noaa.gov/inport/item/69506 (Inventory 2023‐2024). Geospatial data identifying coral reef locations were obtained from UNEP‐WCMC at https://data‐gis.unep‐wcmc.org/portal/home/item.html?id=0613604367334836863f5c0c10e452bf (10.34892/t2wk‐5t34). GFDL Earth System Model 4.1 code was obtained from GitHub at https://github.com/NOAA‐GFDL/ESM4/tree/ESM4 and is archived with Zenodo at https://zenodo.org/record/3836405.
