# Supplementary material for: Potential for Regional Resilience to Ocean Warming and Acidification Extremes: Projected Vulnerability Under Contrasting Pathways and Thresholds
Source: Glob Chang Biol. 2025 Jul 21;31(7):e70360. doi: 10.1111/gcb.70360 (PMC12278047; doi:10.1111/gcb.70360)
Supplement: Supplementary file 1 — Data S1. [file GCB-31-e70360-s001.pdf]

S. Supporting Information for "Potential for regional resilience to ocean warming and acidification extremes: Projected vulnerability under contrasting pathways and thresholds" by E. M. Olson, J. G. John, J. P. Dunne, C. A. Stock, and E. J. Drenkard

S.1 AMM$_{max}$ versus 95th percentile (or AMM$_{min}$ versus 5th percentile)

In the adaptive threshold analyses presented in section 3.2, we made use of an approximate correspondence between the most extreme monthly mean value in a year (AMM$_{max}$ or AMM$_{min}$) and the threshold associated with the most extreme 5% of the distribution (95th or 5th percentile), which we employed as a threshold in the non-adaptive context. In this section, we show that the correspondence is consistent with the relationship between percentile and AMM$_{max}$ that can be derived under the assumption of an idealized sinusoidal seasonal cycle. We then present figures that support the correspondence empirically based on daily and monthly model output.

For a sinusoidal seasonal cycle with peak at time t_{max} in days into a 30-day month, the AMM$_{max}$, or average value over the month containing the maximum value, is given by:

$$AMM_{max}(t_{max}) = \frac{1}{30}\int_0^{30} \cos\left(\frac{2\pi(t - t_{max})}{365}\right) \mathrm{d}t \tag{S.1}$$

AMM$_{max}$ is largest when the peak is centered within the month ($t_{max} = 15$):

$$AMM_{max}(t_{max} = 15) = \frac{1}{30}\int_0^{30} \cos\left(\frac{2\pi(t - 15)}{365}\right) \mathrm{d}t = 0.9889 \tag{S.2}$$

AMM$_{max}$ is smallest when the peak is centered at either end of the month (e.g., $t_{max} = 0$):

$$AMM_{max}(t_{max} = 0) = \frac{1}{30}\int_0^{30} \cos\left(\frac{2\pi(t)}{365}\right) \mathrm{d}t = \frac{1}{30}\frac{365}{2\pi} \sin\left(\frac{2\pi(t)}{365}\right)\Big|_0^{30} = 0.9561 \tag{S.3}$$

Furthermore, for a sinusoidal cycle, the percentile of the underlying distribution represented by AMM$_{max}$ is 100*(1-$\alpha(t_{max})$), where $\alpha(t_{max})$ is the fraction of the year during which AMM$_{max}$ is exceeded:

$$\alpha(t_{max}) = \frac{1}{\pi}\arccos(AMM_{max}(t_{max})). \tag{S.4}$$

Plugging in the quantities from S.1 and S.1, this quantity ranges from 90.5 to 95.3, with an expected value, $< percentile >$ given by

$$< AMM_{max} > = 1/30 \int_0^{30} AMM_{max}(t_{max}) \mathrm{d}t_{max} \tag{S.5}$$

$$< AMM_{max} > = 1/30 \int_0^{30} \frac{1}{30}\frac{365}{2\pi}\left[\sin\left(\frac{2\pi(30 - t_{max})}{365}\right) - \sin\left(\frac{2\pi(0 - t_{max})}{365}\right)\right] \mathrm{d}t_{max} \tag{S.6}$$

$$< percentile > = 100 * (1 - \frac{1}{\pi}\arccos(< AMM_{max}) >)) = 93.3 \tag{S.7}$$

Thus, for an idealized, sinusoidal seasonal cycle, where all values of t_{max} were equally likely, the most probable AMM_{max} would represent the 93.3 percentile.

The actual relationship between percentile and AMM_{max} is determined by the true variable distribution and seasonality, including high-frequency variability and seasonal solar forcing (so that all phases are not necessarily equally likely). Based on daily and monthly model output, we have found empirically that the AMM_{max} or AMM_{min} is an reasonably good proxy for the 95th or 5th percentile value, although the closest match is to the 93/7th or 94/6th percentile (Figure S1).

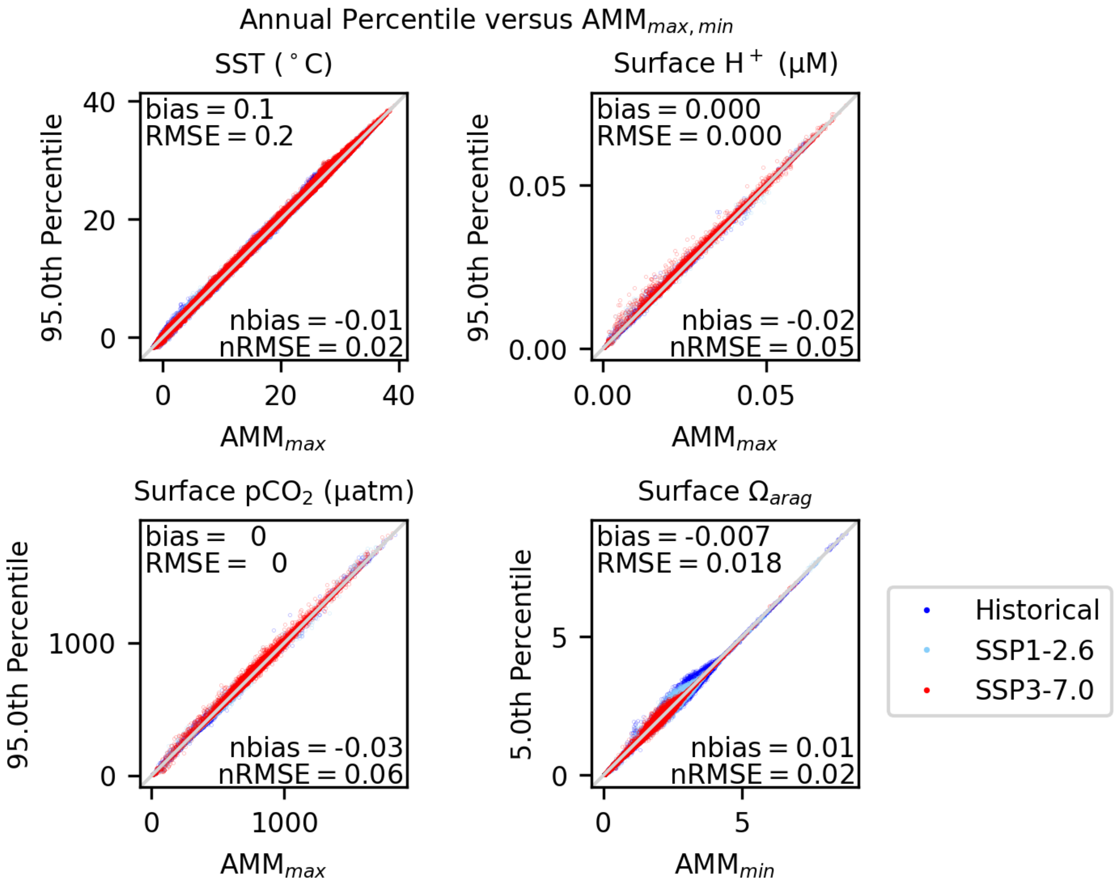

Figure S1: Annual 95th Percentile versus annual maximum or minimum monthly mean, AMM_{max} or AMM_{min}.

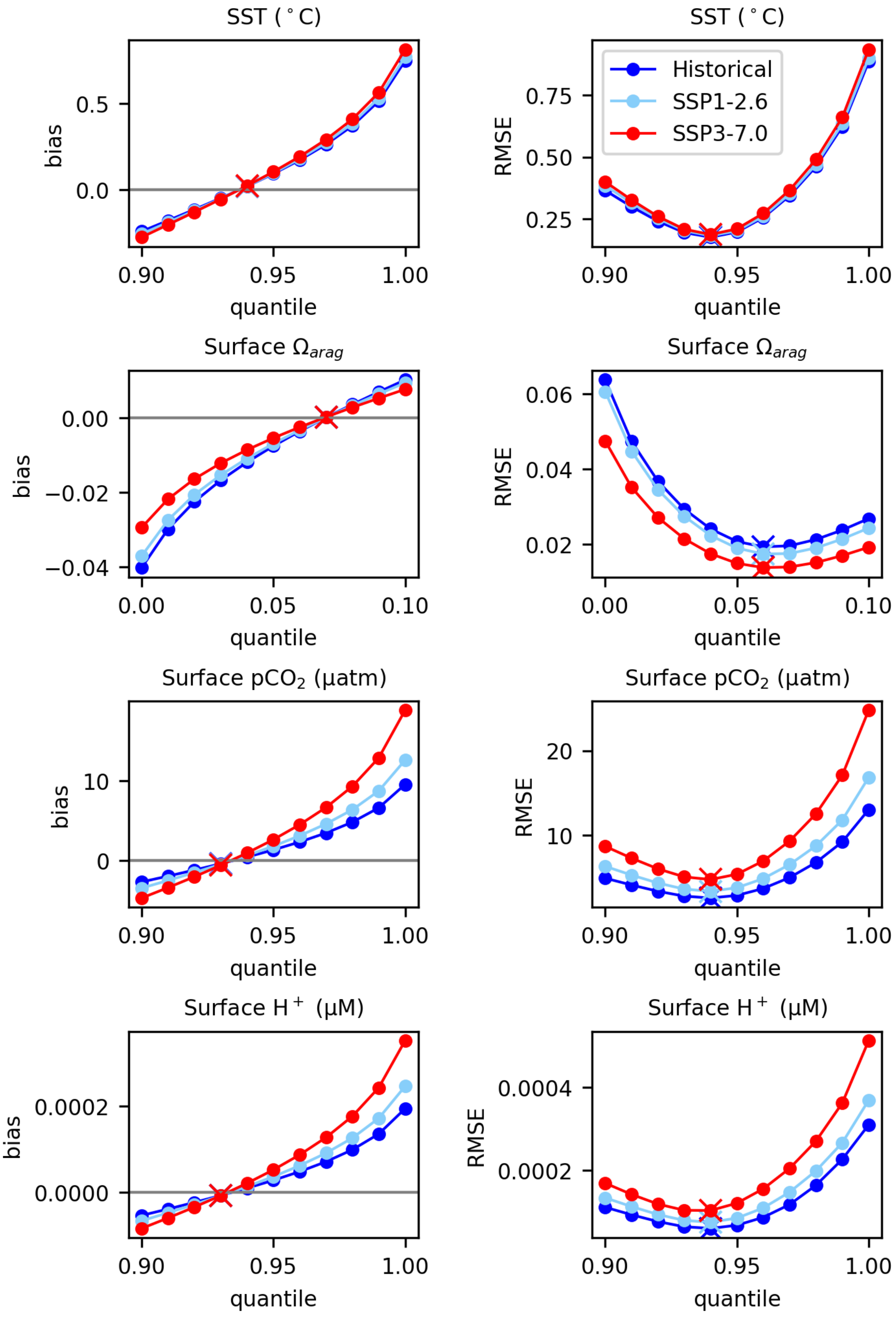

Figure S2: Bias and RMSE of AMM_{max} or AMM_{min} as a predictor of percentiles, from from 90 to 100.

Other Figures

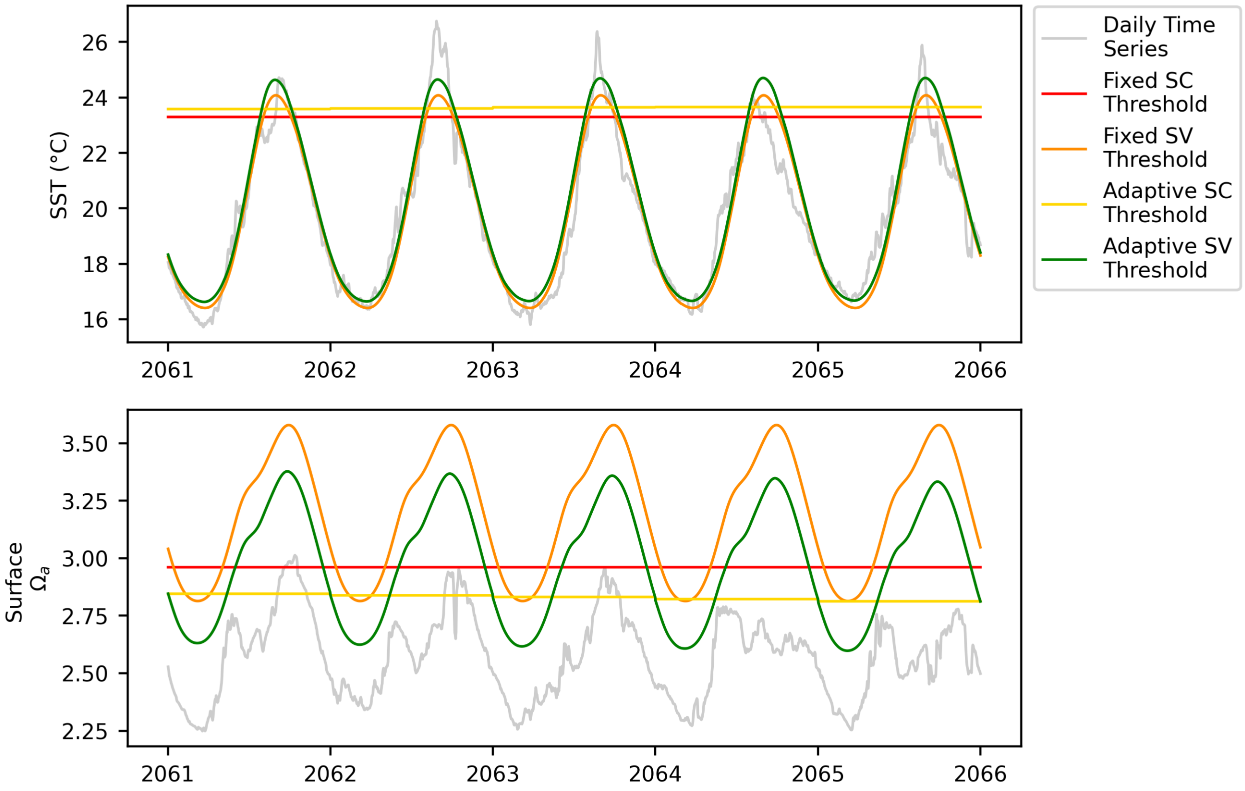

Figure S3: Examples of the percentile-based threshold definitions applied in this study for SST and Ω_a at one location.

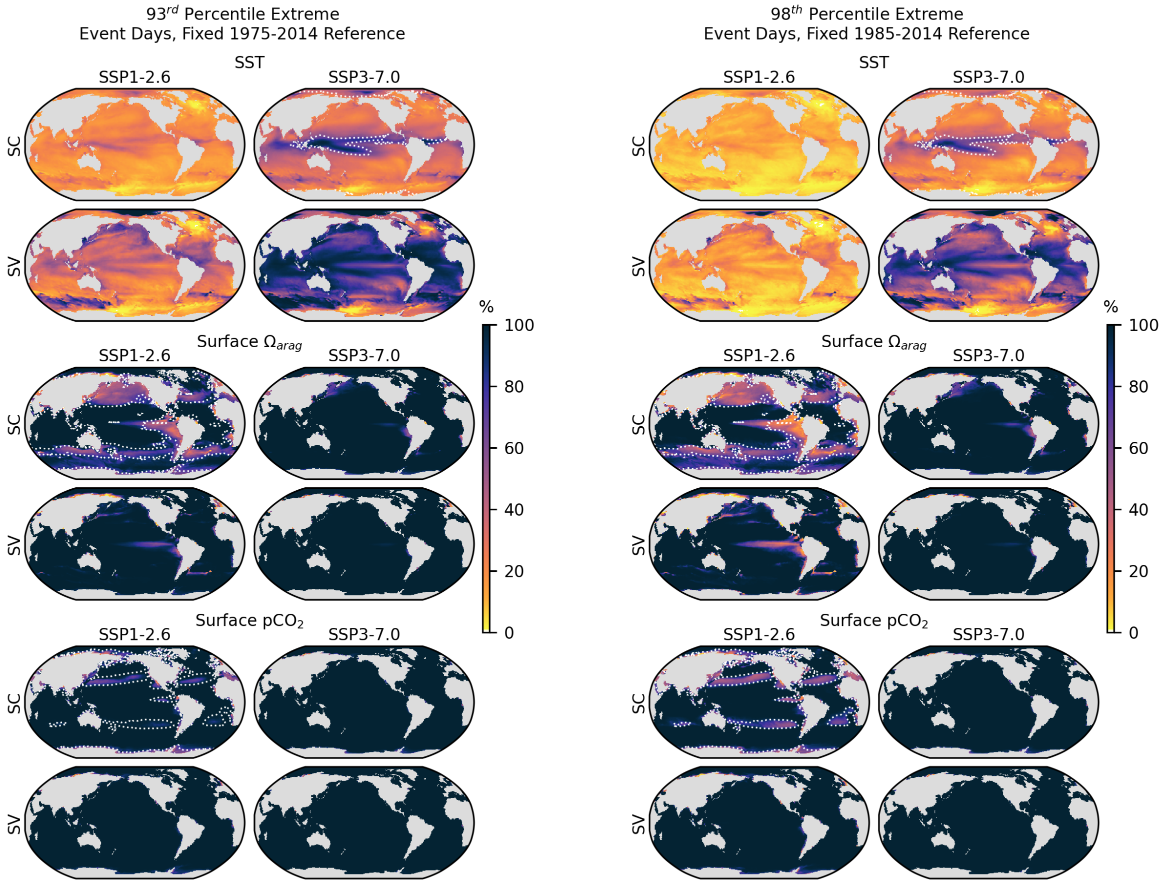

Figure S4: Examples of alternative threshold percentile choices (93, left, and 98, right) demonstrating the consistency in spatial patterns in extreme event frequency. Compare with Figure 1; qualitative regional patterns are insensitive to these choices although the quantity of projected extreme days decreases as the percentile increases.

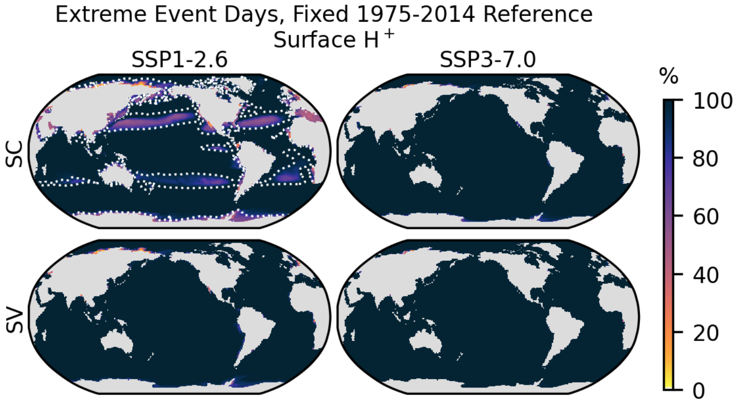

Figure S5: H^+ extreme days relative to fixed reference, as in Figure 1.

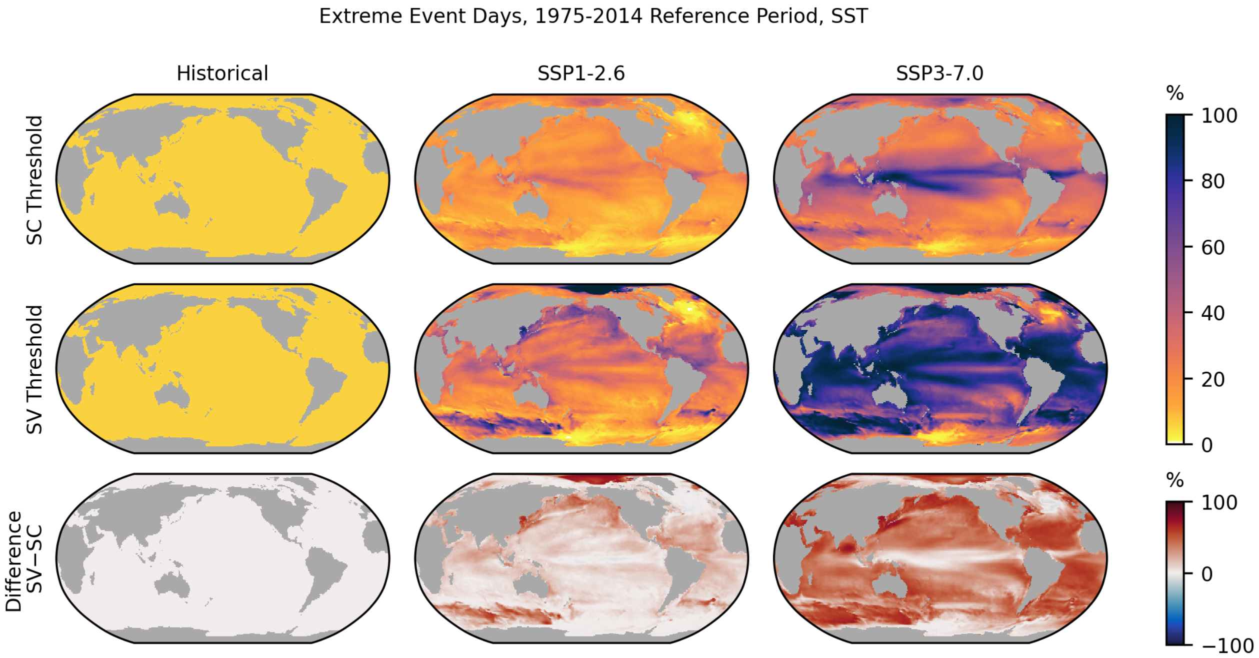

Figure S6: SST extreme days relative to fixed reference, as in Figure 1, with differences plotted explicitly.

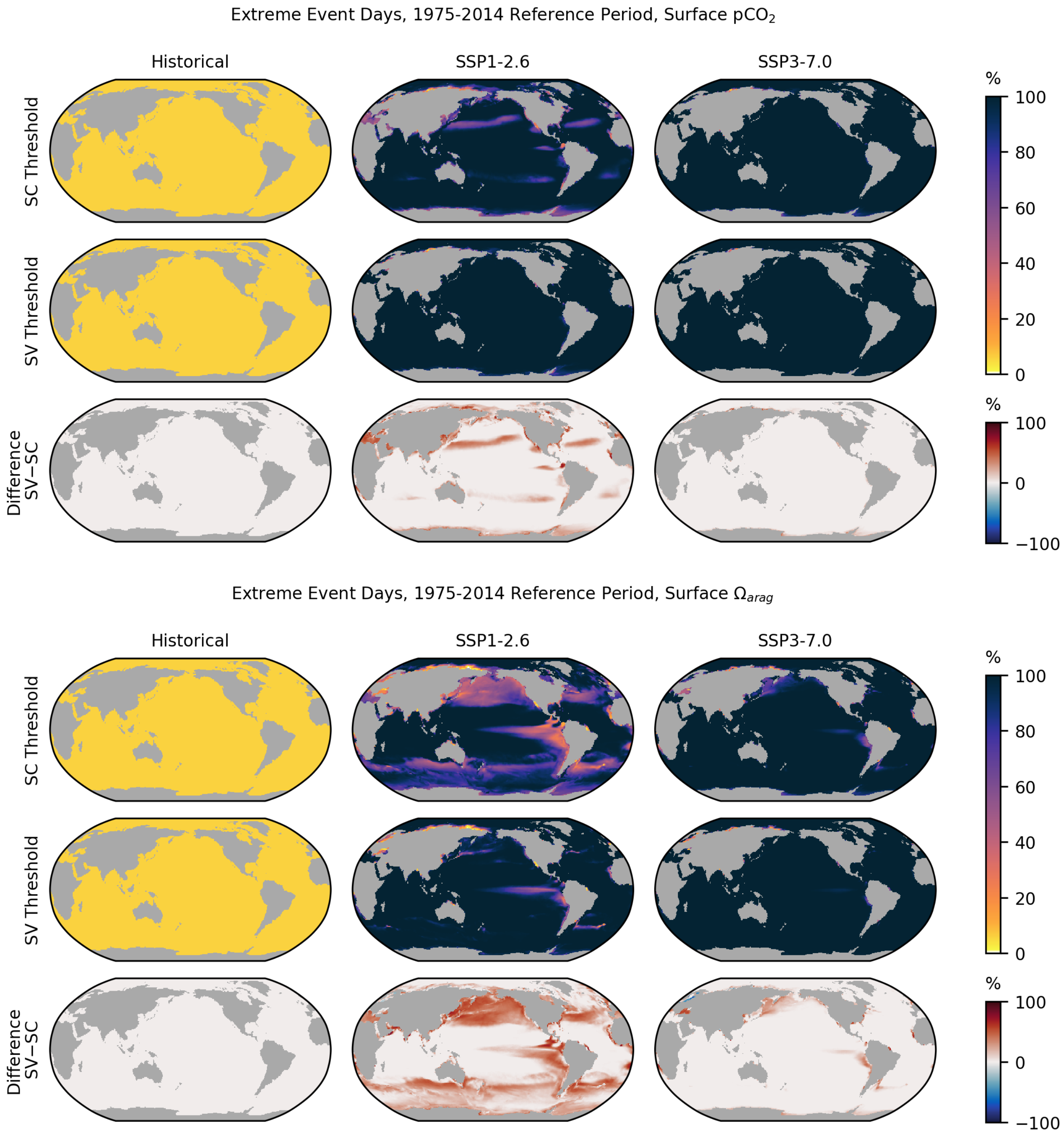

Figure S7: Surface $p\mathrm{CO}_2$ and Ω_a extreme days relative to fixed reference, as in Figure 1, with differences plotted explicitly.

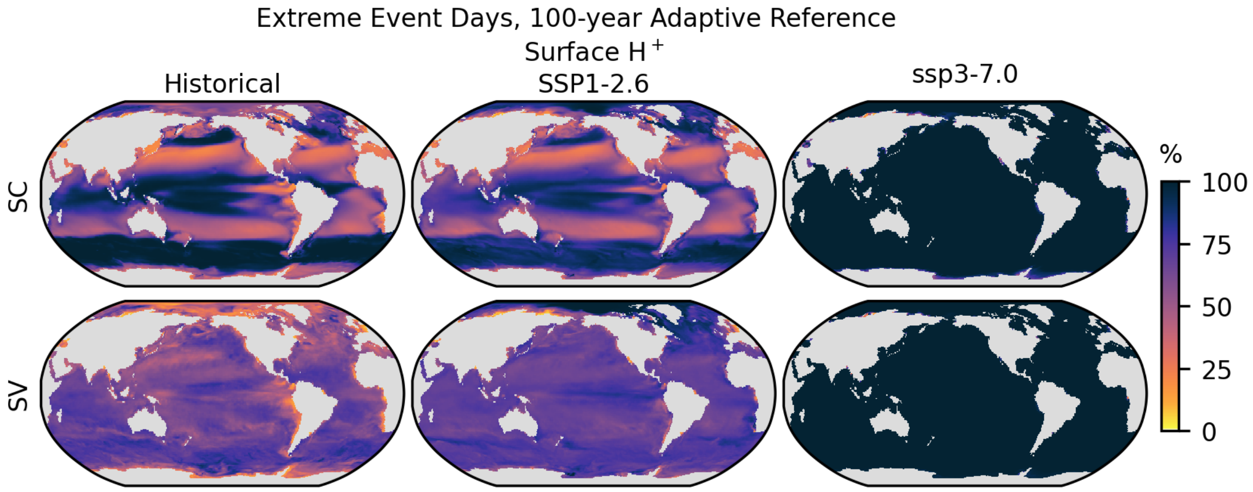

Figure S8: H$^+$ extreme days relative to a 100-year adaptive reference, as in Figure 4.

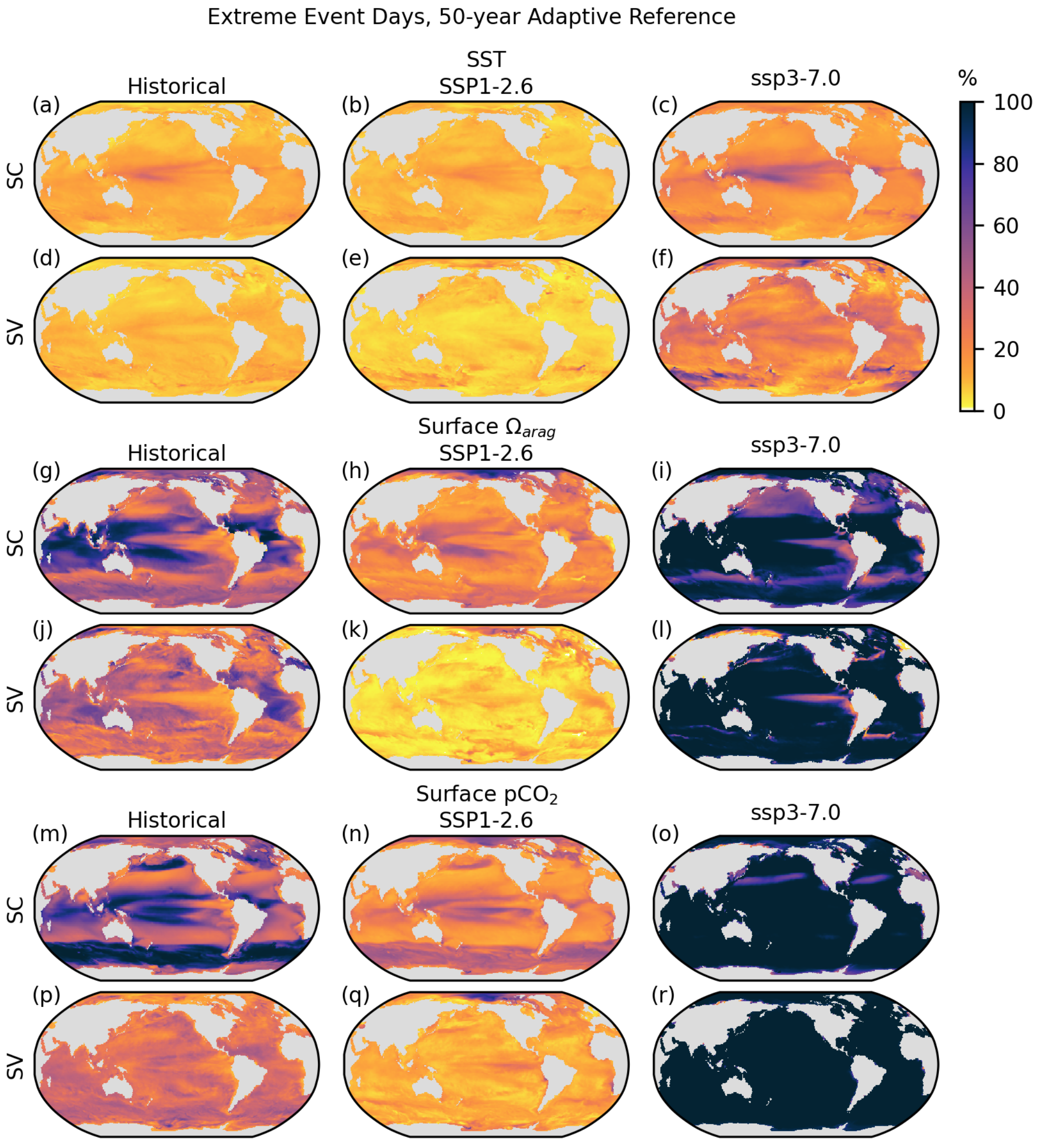

Figure S9: Adaptive thresholds based on 50-year shifting baseline, consistent with more rapid adaptation compared to the 100-year baseline presented in Figure 4.

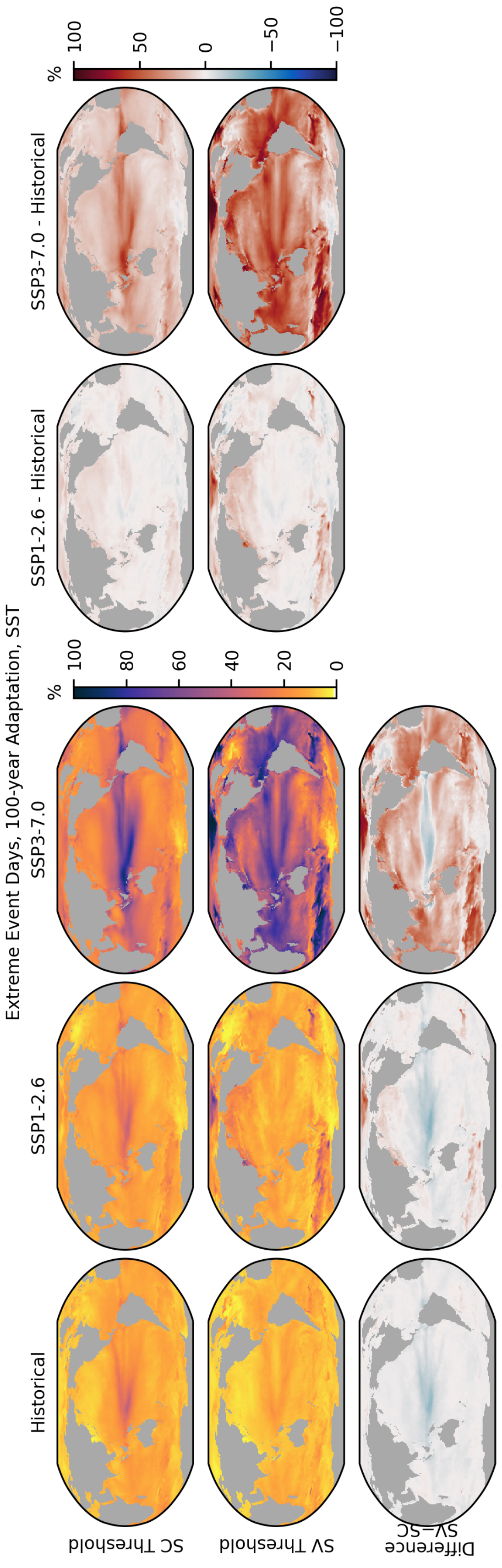

Figure S10: SST extreme days relative to adaptive reference, as in Figure 4, with differences plotted explicitly.

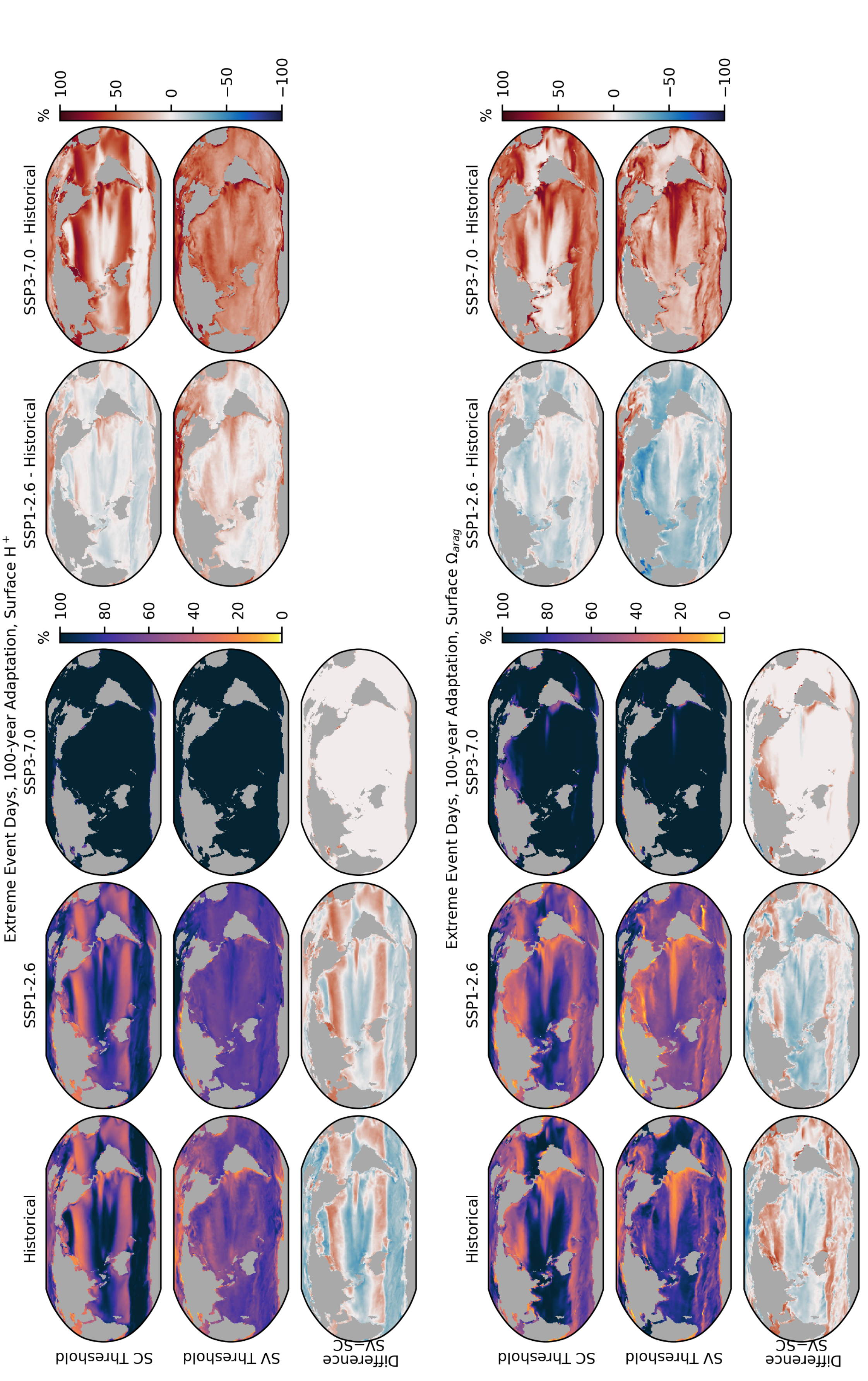

Figure S11: H^+ and Ω_a extreme days relative to adaptive reference, as in Figure 4, with differences plotted explicitly.

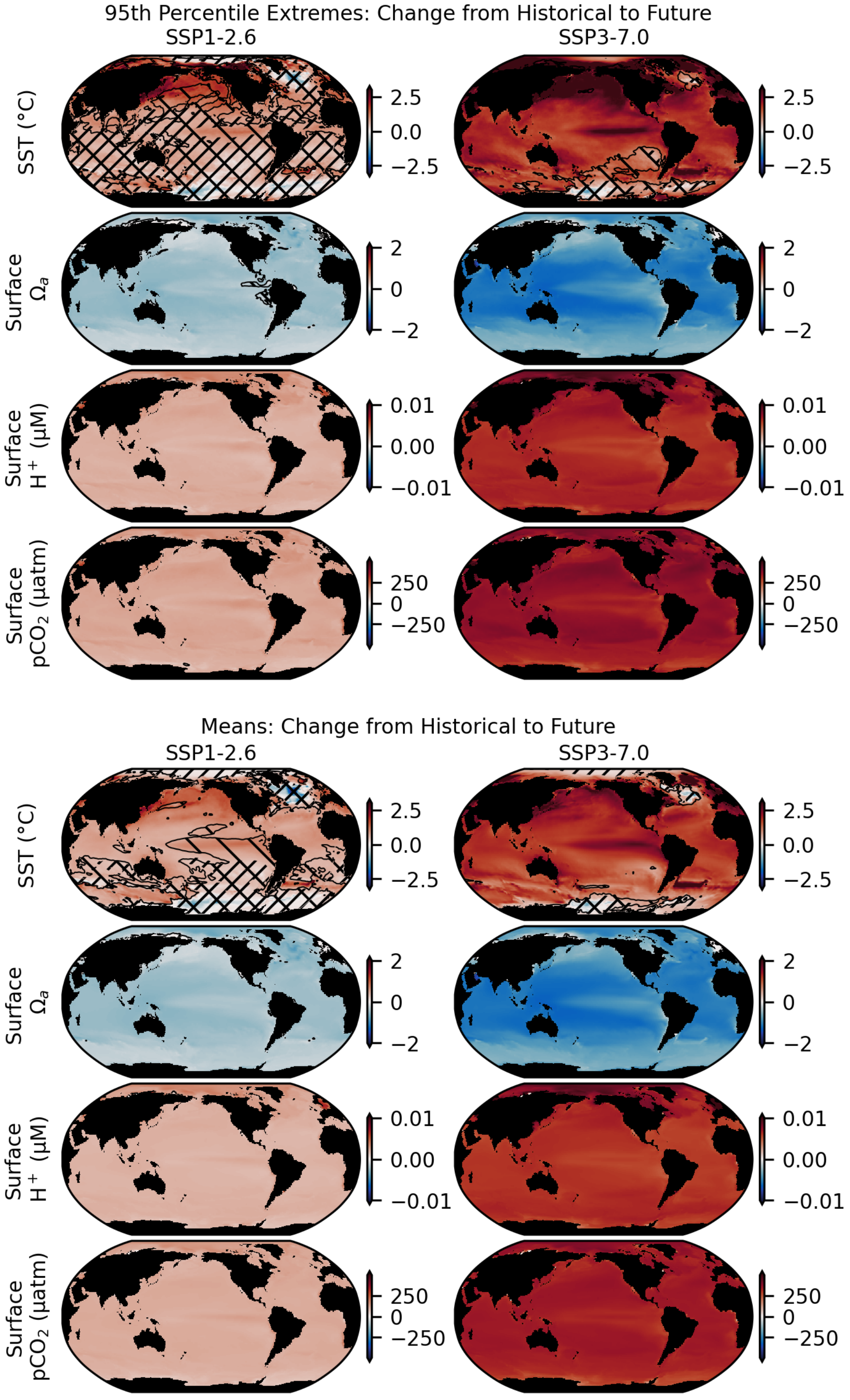

Figure S12: Upper half: historical (1975-2014) to future (2061-2100) change in the 95th (SST, Surface H^+, Surface pCO_2) or 5th (Surface Ω_a) percentile level of each stressor. Hatching indicates amplitude relative to the historical interannual variability of the annual 95th or 5th percentile over the 1975-2014 historical period, with back-slash hatching (\\) where the change does NOT exceed twice the local standard deviation and forward-slash hatching (//) where the change does NOT exceed twice the global mean of the standard deviation. Lower half: The same plots, but for the mean rather than 95th or 5th percentiles.

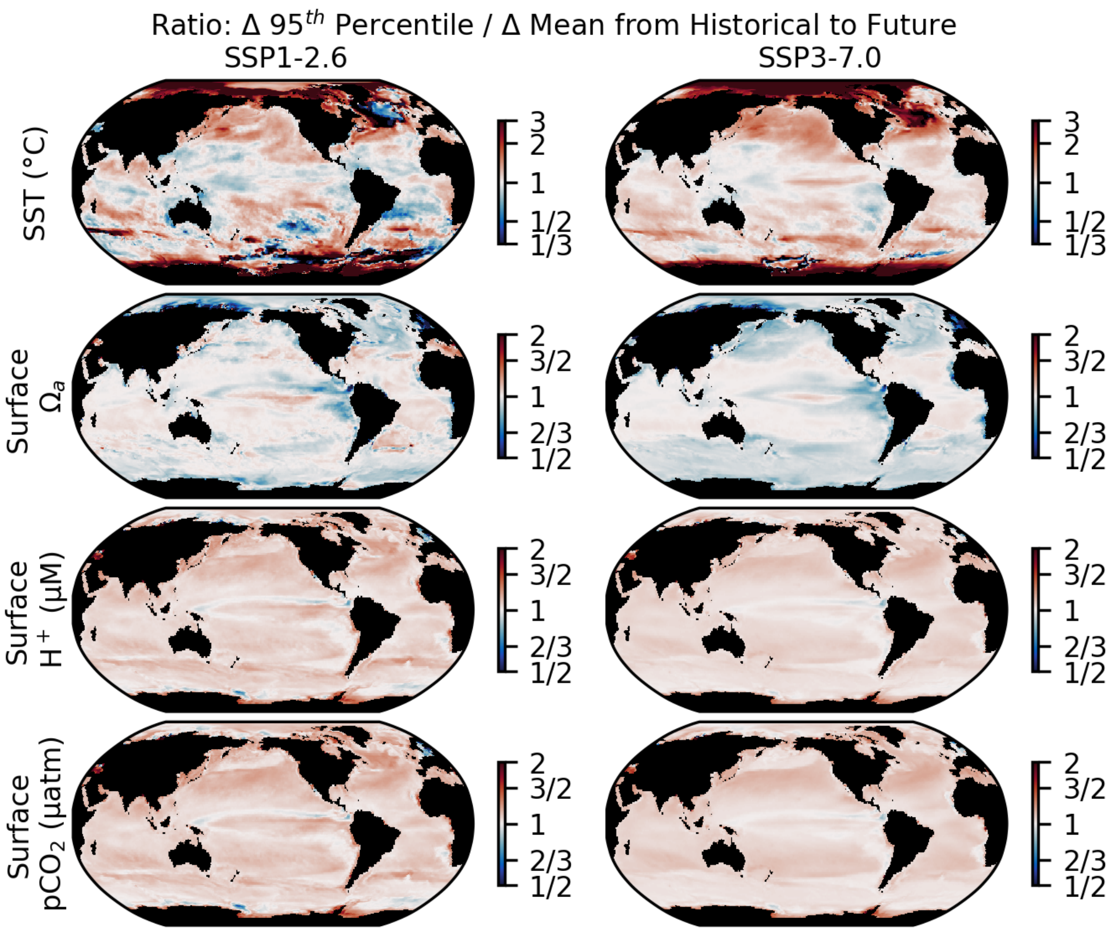

Figure S13: Ratio of difference between change in 95th or 5th percentile and change in mean between approximately 1995 and 2081.

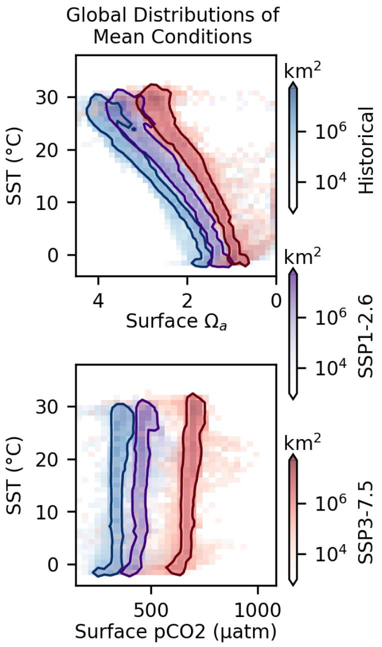

Figure S14: Histograms of mean surface Ω_a conditions (upper left) and SST and surface $p\mathrm{CO}_2$ (lower left) for comparison with Figure 8a and b.

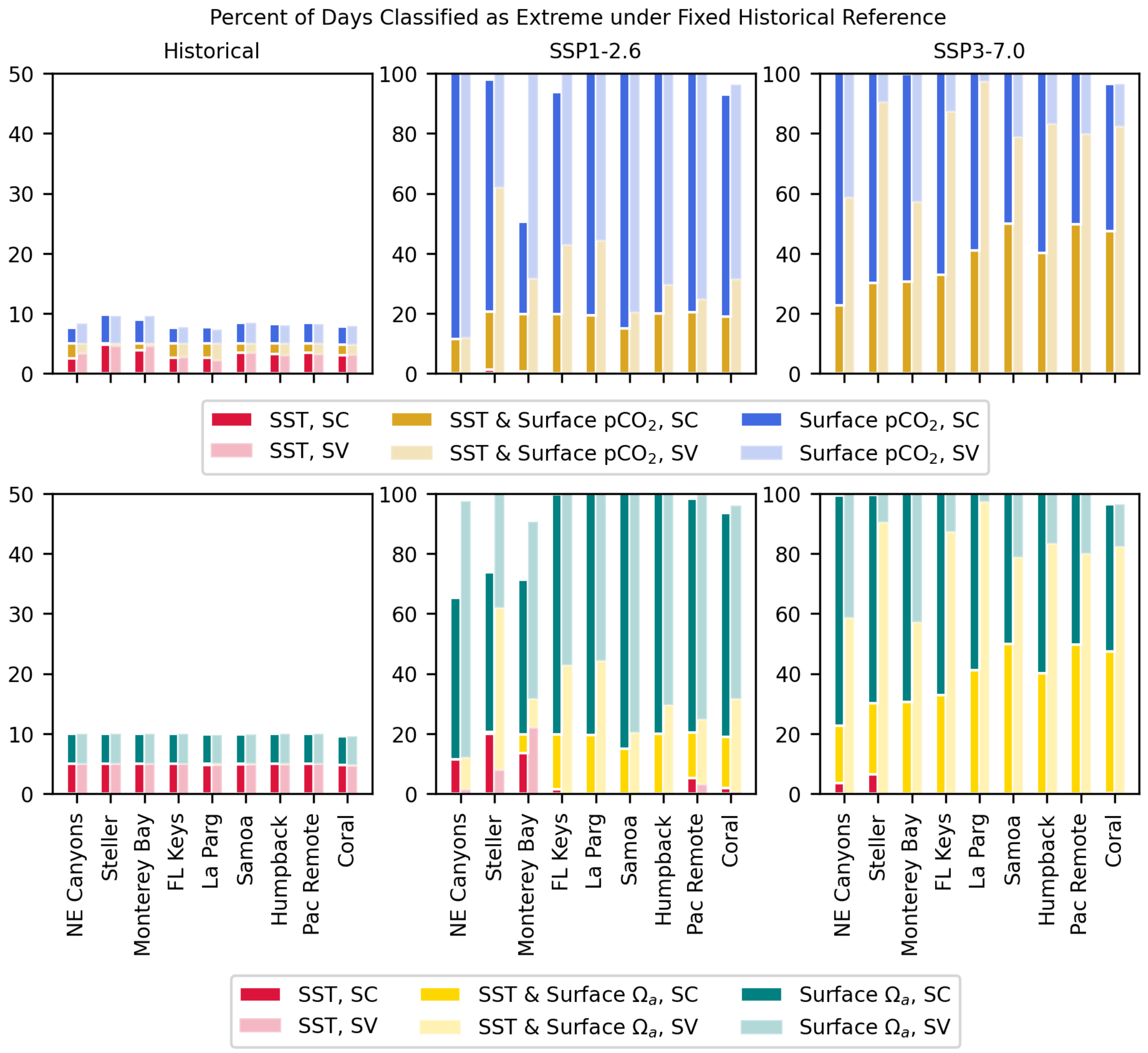

Figure S15: Extreme days at MPA sites based on fixed historical thresholds.

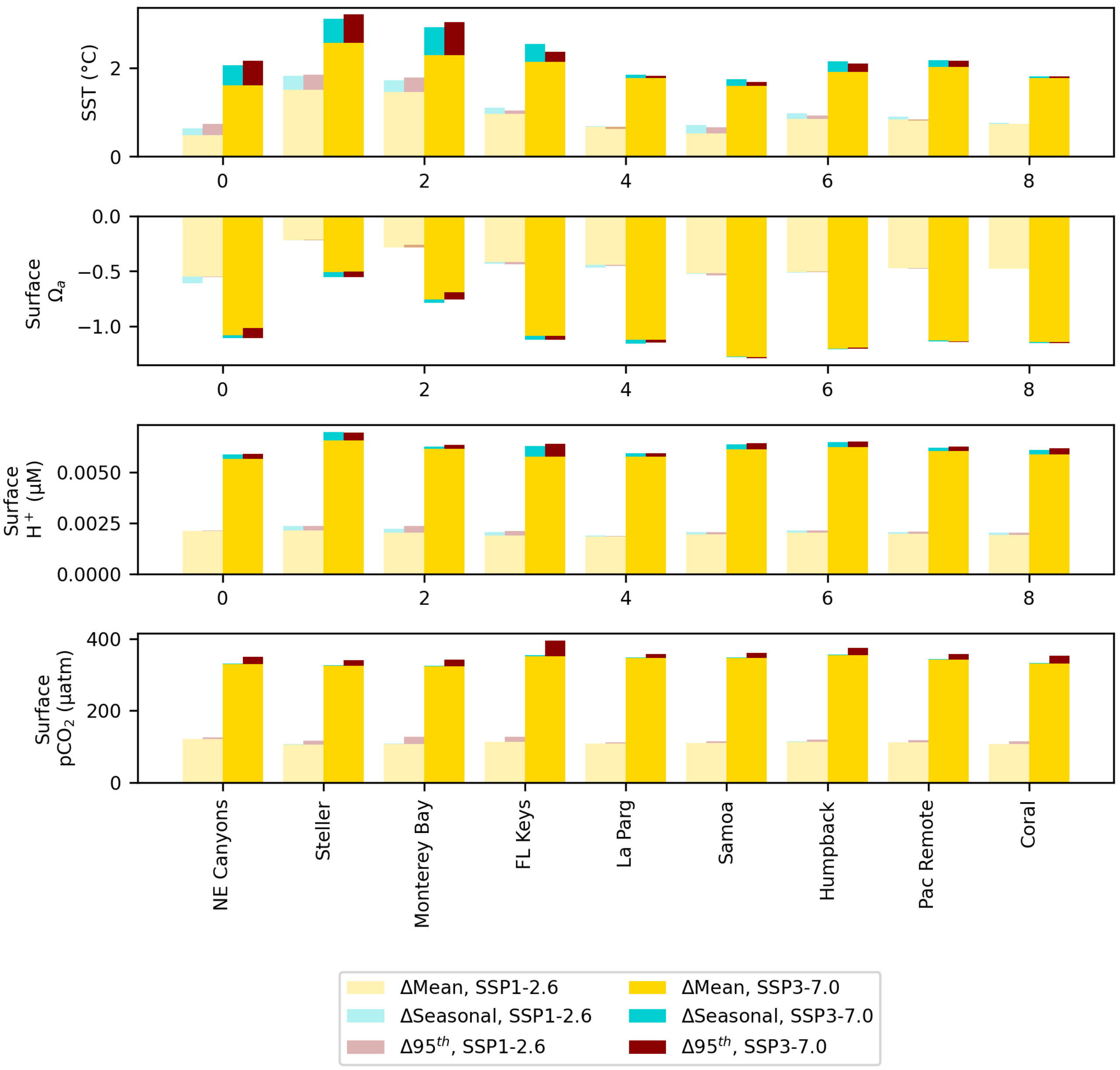

Figure S16: 1995-2081 change in mean, seasonal cycle, and 95th percentile SST, Ω_a, H^+, and pCO_2 at MPA sites. Lighter shaded bars represent projections under SSP1-2.6, and darker shaded bars represent projections under SSP3-7.0.
